# Rapid isolation of antigen-specific B-cells using droplet microfluidics†

**DOI:** 10.1039/d0ra04328a

**Published:** 2020-07-20

**Authors:** Ruihua Ding, Kuo-Chan Hung, Anindita Mitra, Lloyd W. Ung, Daniel Lightwood, Ran Tu, Dale Starkie, Liheng Cai, Linas Mazutis, Shaorong Chong, David A. Weitz, John A. Heyman

**Affiliations:** John A. Paulson School of Engineering and Applied Sciences, Harvard University 11 Oxford Street Cambridge MA 02138 USA jheyman@g.harvard.edu +1-760-884-9961; Department of Chemistry and Chemical Biology, Harvard University Cambridge MA 02138 USA; UCB Pharma 216 Bath Road Slough SL1 3WE UK; CAS Key Laboratory of Systems Microbial Biotechnology, Tianjin Institute of Industrial Biotechnology, Chinese Academy of Sciences Tianjin 300308 P.R. China; Department of Materials Science and Engineering, University of Virginia 395 McCormick Road Charlottesville Virginia 22904 USA; Department of Chemical Engineering, University of Virginia 395 McCormick Road Charlottesville Virginia 22904 USA; Vilnius University, Institute of Biotechnology Vilnius LT 01257 Lithuania; New England Biolabs 240 County Road, Ipswich MA 01938 USA; Arbor Biotechnologies 790 Memorial Dr. Cambridge MA 02139 USA; Department of Physics, Harvard University 29 Oxford Street Cambridge MA 02138 USA weitz@seas.harvard.edu +1-617-496-2842; SphereBio 18 Laurel TR, Unit 2 Somerville MA 02143 USA

## Abstract

Monoclonal antibodies are powerful tools for scientific research and are the basis of numerous therapeutics. However, traditional approaches to generate monoclonal antibodies against a desired target, such as hybridoma-based techniques and display library methods, are laborious and suffer from fusion inefficiency and display bias, respectively. Here we present a platform, featuring droplet microfluidics and a bead-based binding assay, to rapidly identify and verify antigen-binding antibody sequences from primary cells. We used a defined mixture of hybridoma cells to characterize the system, sorting droplets at up to 100 Hz and isolating desired hybridoma cells, comprising 0.1% of the input, with a false positive rate of less than 1%. We then applied the system to once-frozen primary B-cells to isolate rare cells secreting target-binding antibody. We performed RT-PCR on individual sorted cells to recover the correctly paired heavy- and light-chain antibody sequences, and we used rapid cell-free protein synthesis to generate single-chain variable fragment-format (scFv) antibodies from fourteen of the sorted cells. Twelve of these showed antigen-specific binding by ELISA. Our platform facilitates screening animal B-cell repertoires within days at low cost, increasing both rate and range of discovering antigen-specific antibodies from living organisms. Further, these techniques can be adapted to isolate cells based on virtually any secreted product.

## Abstract 2

We use a droplet-microfluidics-based platform to rapidly identify and isolate individual primary cells that secrete desired antibodies. We then retrieve the antibody-encoding sequences and create recombinant antibodies that bind the target protein.

## Graphical abstract

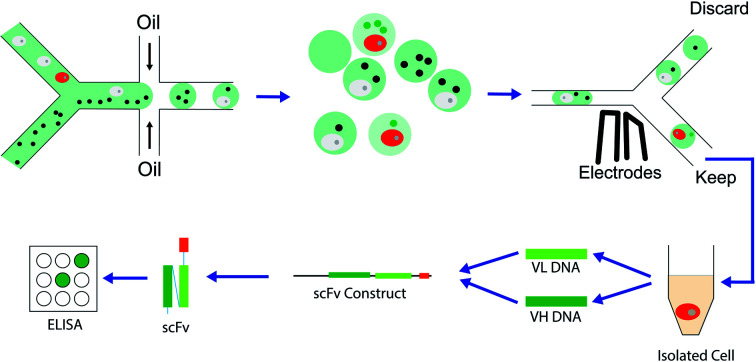

## Introduction

Biological research and therapeutic development increasingly rely on monoclonal antibodies;^1–4^ however, current methods to generate high-affinity, high-specificity monoclonal antibodies do not adequately meet this demand. Primary B-cells, the main source of monoclonal antibody sequences, are short lived and expand poorly in culture,^5^ making high-throughput screening for single cells producing desired IgG challenging. This problem is partially overcome by conventional hybridoma technology in which B-cells are fused with myeloma cells to generate immortal hybridoma cells that secrete B-cell-encoded antibody.^6^ The hybridoma cells are then serially diluted and cultured into clonal cell populations that secrete sufficient antibody for binding analysis. This selection process is laborious and time-consuming, limiting throughput. Recently, droplet-based methods have been developed to massively increase the throughput of hybridoma screening.^7,8^ However, the fusion step remains inefficient, and a large portion of the antibody-producing cells are not immortalized.^9^

Alternative methods use display libraries.^10–13^ Here the antibody heavy and light chain sequences from a large population of B-cells are randomly paired and assembled into an expression library. The library is then expressed on the surface of host cells or on phage particles and the cells or phage that display antigen-specific antibodies are selected by panning against an immobilized target protein. Recently, several groups have addressed a drawback of display methods, the unnatural combinatorial pairing of heavy and light chains during library construction,^14^ by performing single-cell reverse-transcription PCR in microfluidic droplets. This maintains the native pairing of heavy and light chains, and impressive antibody isolations have been reported.^15–18^ Another potential shortcoming of display methods, reduced accessibility of the displayed molecules, has been addressed by barcoding potential binding proteins with small peptide tags (11–15 kDa) that can be identified by mass spectrometry.^19^ This method eliminates the need for display on bulky objects such as phage or yeast cells, but it requires a high level of expertise and specialized equipment. Overall, display methods all may incur diversity loss during library preparation, and for some applications, binders isolated as full-length IgGs may be preferable to those isolated through display methods.

These issues can be addressed by directly isolating individual cells that secrete a desired antibody, so that the IgG-encoding genes can be identified and used to generate recombinant antibodies. Published methods include droplet-microfluidics platforms using FRET-based detection,^20^ and platforms that perform single-cell assays in microwells/microchambers^21–25^ or in microcapillary arrays.^26–28^ These all enable rapid identification of single cells that secrete desired antibodies; however, they typically require highly specialized instruments and assay components (ESI Appendix Table S1†). An elegant microscopy-based method to screen a very large number of gel-encapsulated cells has been described, but the hand-picking step limits the number of cells recovered.^29^ Thus, there is no single optimal method to rapidly analyze antibodies produced by single primary IgG-secreting B-cells.

In this study, we demonstrate a droplet microfluidics-enabled process to isolate and functionally verify paired VH- and VL-genes from individual antibody-secreting cells. In final proof-of-principle experiments, we isolate rare cells that secrete anti-TNF-α antibody, constituting ∼0.2% of the input, from a population of non-immortalized splenocytes derived from TNF-α immunized rat. We use an in-droplet, bead-based fluorescence concentration assay to identify droplets containing single cells that secrete target-binding antibody, and then perform dielectrophoretic sorting to select these droplets at high throughput. We use single-cell reverse-transcription PCR to recover correctly paired heavy- and light-chain gene sequences. We then use cell-free synthesis to synthesize scFv-format versions of the secreted antibodies and find that the isolated gene sequences encoded TNF-α-binding heavy- and light-chain variable regions. These results demonstrate that droplet-microfluidics is a powerful tool to generate target-binding monoclonal antibodies and will speed development of antibody-based reagents and therapeutics. We note that, subsequent to our original submission, Gerard *et al.*^30^ described a similar-in-principle droplet-based method to isolate desired antibody-secreting cells. This method featured an elegant use of magnetically alignable capture beads, which allowed the authors to perform affinity analysis of cell-secreted antibodies prior to sorting. The two platforms differ in some respects, and aspects of each might be useful in specific applications (see Table S1† for discussion).

## Results

**Fig. 1:**
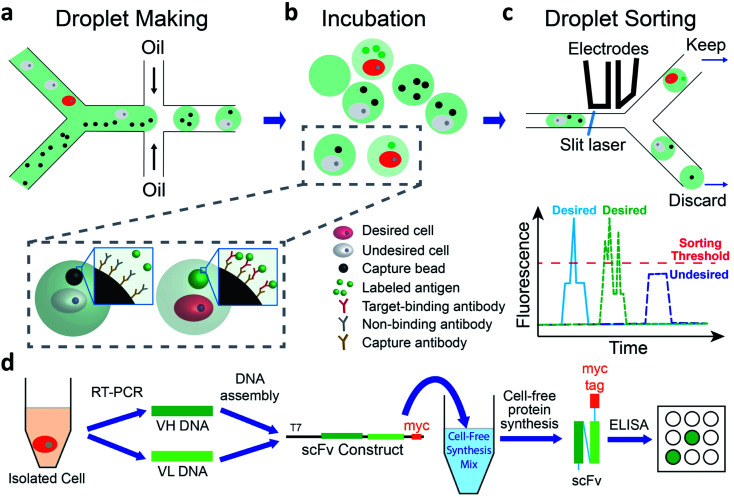
Overview of method. (a–c) In-droplet binding assay and sorting. (a) Cells, capture beads, and Alexa-488 labeled antigen are co-encapsulated into droplets using a co-flow droplet making device. (b) The droplets are incubated at 37 °C so that cells secrete antibody. Desired cells (red) secrete antibodies that bind to Alexa-488 labeled antigen, leading to concentration of fluorophore onto the surface of the bead. Undesired cells (light gray) secrete irrelevant antibodies and fluorescent signal is not concentrated onto the bead. (c) Incubated droplets are loaded into a droplet sorting device and sorted based on the presence of the fluorescent bead, detected as a peak (one capture bead, blue trace), or potentially multiple peaks (multiple capture beads, green trace) above droplet fluorescence pedestal. Desired droplets are kept for further analysis. (d) Generate and test recombinant versions of desired antibodies. RT-PCR is performed on individual droplet-sorted cells to isolate VH- and VL-encoding DNA. These DNAs are assembled into constructs to direct cell-free synthesis of myc-tagged scFv proteins. ELISA is used to test scFv protein binding to a panel of plate-immobilized proteins.

We use a droplet microfluidics-based platform to isolate and functionally verify paired VH- and VL-genes directly from antibody-secreting cells (Fig. 1). We co-encapsulate cells, capture beads and fluorescent antigen in picoliter-sized droplets at more than 2000 Hz (Fig. 1a and ESI Appendix Fig. S1†). We then incubate droplets for cells to secrete antibodies, which are captured by the capture beads. If the antibody binds to the fluorescently labeled antigen, the fluorescence at bead surface increases (Fig. 1b). We use a sorting device (ESI Appendix Fig. S2†) mounted on a droplet detection and sorting instrument to screen the droplets at up to 100 Hz and isolate droplets having highly fluorescent capture beads^7,31^ (Fig. 1c). The passive size filter^31^ in our sorting device (ESI Appendix Fig. S2,† element c) shunts abnormally large (merged) droplets into the waste, preventing them from shearing at the sorting junction. This dramatically reduces the flow of droplet fragments, which can contain undesired cells, into the sort and greatly increases sorting accuracy. We use single cell reverse transcription PCR (RT-PCR) to recover correctly paired VH- and VL-genes. We then use cell-free synthesis to produce scFv-format versions of the desired secreted antibodies and Enzyme Linked Immunosorbent Assays (ELISA) to analyze their binding (Fig. 1d).

We performed the fluorescent sandwich assay in bulk, using high cell and bead concentrations to mimic conditions in ∼40 pL droplets, to confirm that capture bead fluorescence is antibody-dependent. When we mixed anti-mouse IgG-coated capture beads and Alexa Fluor™ 488-labeled human tumor necrosis factor alpha (TNF-α) with either buffer only or hybridoma 9E10 cells that secrete anti-cMyc antibody (abbreviated anti-cMyc cells below), the fluorescent intensity of beads was at background level (ESI Appendix Fig. S3a†). However, when the capture beads and Alexa Fluor™ 488-TNF-α protein were mixed with anti-TNF-α antibody-secreting hybridoma 357-101-4 cells (abbreviated anti-TNF-α cells below), the beads became highly fluorescent (ESI Appendix Fig. S3b†). During a 12 h incubation at 37 °C, the bead fluorescence reached a plateau after ∼20 min (ESI Appendix Fig. S3c†) and did not diminish significantly thereafter.

**Fig. 2:**
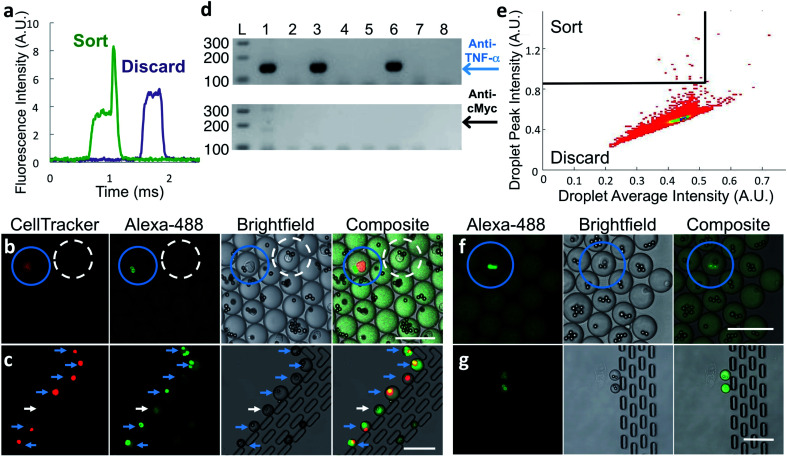
Sorting of hybridoma and primary cells. (a) Representative PMT traces of droplets containing (green trace) or lacking (purple trace) a fluorescent bead. (b–d) Hybridoma sorting. Red-stained anti-TNF-α-secreting M357 cells were combined at a 1 : 1000 ratio with unlabeled anti-cMyc secreting cells and encapsulated into droplets with capture beads and Alexa 488-labeled TNF-α protein. (b) Droplets imaged prior to sorting. Blue circle indicates desired droplet with red-stained anti-TNF-α cell and highly fluorescent beads. Droplet circled with white dashed line contains an undesired anti-cMyc-secreting cell and the beads are not fluorescent. (c) Seven sorted droplets collected in observation trap. Six contain bright beads and a cell-tracker-stained anti-TNF-α cell, indicating correct sorting (blue arrows). One droplet was sorted due to the presence of a fluorescent cell (white arrow). (d) Representative gel-electrophoresis results from RT-PCR performed on individual sorted droplets dispensed into tubes. Amplification reactions specific for anti-TNF-α and anti-cMyc IgG heavy-chain coding DNA were electrophoresed on the upper and lower agarose gels, respectively. Positions of the respective 147 and 202 bp products are indicated by the blue arrow and black arrows. Only anti-TNF-α product was generated from three of the RT-PCR tubes (lanes 1, 3, 6); no product was generated from the other wells. (e–g) Rat splenocyte results. (e) Typical sorting heat map showing gating region (top-left section). (f) Fluorescence microscopy image of droplets prior to sorting. Droplet highlighted with blue circle contains a desired cell and fluorescent beads. (g) Image of sorted droplets in observation trap shows highly fluorescent beads in the droplets. Scale bars: 75 μm.

To verify that antibody secreted by single cells in droplets generates a signal suitable for identifying and sorting droplets, we co-encapsulated anti-TNF-α cells at less than one cell per droplet along with capture beads and labeled TNF-α protein and analyzed the droplets by microfluidic sorting instrument. A fraction of droplets showed distinct above-pedestal fluorescence peaks (Fig. 2a) due to the presence of a bright bead. This strongly suggests that the in-droplet binding assay sensitivity is sufficient to detect antibodies secreted from single cells.

We determined the accuracy of the sorting platform using samples composed of a mixture of two hybridoma cell lines, anti-TNF-α cells labeled with CellTracker™ DeepRed dye mixed at a 1 : 1000 ratio with unlabeled anti-cMyc cells. We generated droplets using these mixed cells, capture beads, and Alexa Fluor™ 488-labeled TNF-α protein. After incubation, we imaged a small fraction of the droplets. As expected, Alexa Fluor™ 488-labeled TNF-α protein is concentrated onto capture beads only in the small population of droplets that also contains fluorescently labeled anti-TNF-α cells (Fig. 2b, blue circles). In droplets with all other component combinations, including those with an anti-cMyc cell and capture beads, fluorescence was uniformly distributed (Fig. 2b, dashed white circle).

We sorted droplets having a fluorescence peak above the pedestal (Fig. 2a) and imaged a small fraction of the sorted droplets.^31^ In the shown example, six of the seven droplets collected in the observation trap contained red-stained cells with fluorescent beads, indicating they are desired droplets (Fig. 2c, blue arrows). One droplet was sorted due to the presence of a cell exhibiting fluorescence similar to that of a bead; the reasons for this are unknown (Fig. 2c, white arrows). We cannot observe large numbers of droplets this way because droplets shrink over time in the trap, likely due to water evaporation through PDMS.

To further test sorting accuracy, we performed hybridoma-clone-specific RT-PCR to analyze cells released from sorted droplets. In two experiments, we sorted a total of 127 droplets into a tube containing “dilution” droplets and dispensed the droplet mixture into 228 PCR tubes. We then broke the droplets and performed nested RT-PCR. To simplify primer design, we used a template-switching method, optimized for use with single cells. For the RT and first-round PCR we used reverse primers that anneal to mRNA encoding the heavy-chain-constant region of both anti-TNF-α and anti-cMyc cells. We then performed nested PCR using reverse primers specific for either anti-TNF-α or anti-cMyc cell heavy-chain cDNA to detect the presence of each cell type (see Materials and methods and Fig. S4 in ESI† Appendix for details). Representative DNA electrophoresis results from eight tubes show an anti-TNF-α-specific product generated from three tubes; no product was generated from the other five tubes (Fig. 2d). Overall, an anti-TNF-α-specific PCR product was generated for 82 of the 228 tubes, approximately the number predicted assuming perfect sorting and Poisson distribution during droplet dispensing into RT-PCR wells. In contrast, the anti-cMyc-specific PCR product was generated from only two tubes, and one of these also contained an anti-TNF-α cell. We did not recover all the positive cells due to stringent gating and some droplets not having a bead. The RT-PCR results are summarized in Table 1. Taken together, these results demonstrate that the in-droplet binding assay is antigen-specific and can detect antibodies secreted from single cells, and that the sorting platform is highly accurate, being able to isolate positive cells that are present at 0.1% of the total population with <2% false positives.

**Table 1:** Hybridoma sorting results

	Sort 1	Sort 2	Total
Number of droplets screened	600 000	750 000	1.35 × 10^6^
Number of droplets sorted	40	87	127
Number of wells droplets were dispensed into	49	179	228
Number of wells expected to contain an anti-TNF-α cell (by Poisson distribution)	27	69	96
Number of wells PCR-positive for anti-TNF-α cell	21	61	81
Number of wells PCR-positive for anti-c-Myc cell	0	1	1
Number of wells PCR-positive for both anti-TNF-α cell and anti-c-Myc cell	0	1	1

With the platform verified with hybridoma cells, we used it with frozen stocks of *in vitro*-activated primary splenocytes from rats immunized with human TNF-α protein. Cells that secrete anti-target antibodies comprise a small percentage of these input populations. To ensure that the binding assay sensitivity is sufficient to detect antibodies secreted by single primary B-cells, we encapsulated splenocytes and assay reagents, incubated for 2 h at 37 °C, and then inspected the droplets using confocal microscopy. Droplets containing a cell and brightly fluorescent bead(s) (Fig. 2f, blue circles) were extremely rare; however, their presence strongly suggested that the binding assay sensitivity was sufficient for droplet sorting.

To sort droplets containing cells secreting antigen-binding antibodies, we plotted droplet average fluorescence intensity *versus* peak fluorescence intensity, using average droplet intensity to gate out debris. Desired droplets exhibited a broad range of maximum peak intensity, likely due to the fluorescent beads being in different focal planes during fluorescence detection, and because cells secrete antibodies of differing affinity and at different levels. Typically, we set a stringent sorting threshold to maximize purity; a less-stringent threshold may lead to selection of additional desired droplets, but at reduced purity. A typical heatmap with a stringent threshold is shown in Fig. 2e. We observed a small fraction of the sorted droplets using the observation trap. The majority contained a cell and highly fluorescent bead(s). We also observed droplets containing only a fluorescent cell. A typical image of sorted droplets is shown in Fig. 2g.

In a representative sorting experiment using once-frozen activated splenocytes from a single rat inoculated with TNF-α, we isolated 38 droplets from a population of 380 000 droplets and dispensed them into 104 tubes. Using RT-PCR, we recovered at least one antibody chain from 21 of the wells, indicating a secreting cell was in each of those wells. We recovered both chains from four of the wells, enabling subsequent testing of antibody binding. We attribute our inability to recover antibody genes from each sorted droplet to occasional false sorting positives and to mRNA degradation in these delicate cells during the sorting process; use of fresh, never-frozen input cells will likely result in improved PCR recovery rates.

We performed five sorting experiments using once-frozen activated splenocytes from different TNF-α-inoculated rats. We obtained heavy- and light-chain antibody sequence pairs from 30 of the dispensed tubes (ESI Appendix Dataset S1†). We analyzed these sequences using the International ImMunoGeneTics Information System (IMGT) database^32^ and found that our isolated IGHV-regions fall most commonly into the IGHV5 and IGHV2 families. Additionally, 7 of the 13 rat IGHV families are represented, suggesting that our platform has no significant bias towards particular IGHV families.

**Fig. 3:**
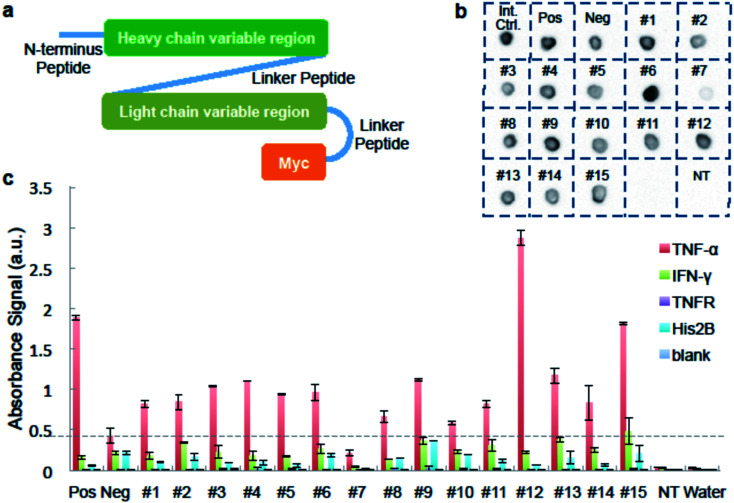
Binding analysis of isolated antibody sequences. (a) A schematic of the scFv constructed from IgG sequences from isolated cells. For panels b and c, we generated scFv proteins in cell-free synthesis reactions programmed with: no DNA template; a “Positive” scFv derived from the TNF-α-binding antibody Adalimumab; a “Negative” scFv that should not bind to TNF-α; and each of fifteen sorting-derived scFv constructs (#1–15). (b) Image of dot blot verifying protein expression. Cell-free synthesis reactions were directly dotted on the membrane. Synthesized scFv was detected using anti-c-Myc antibody conjugated to HRP. “Int. Ctrl.” = internal control, a sample known to be detected by anti-c-Myc dot blot. (c) ELISA results of synthesized scFv against different antigens. scFv reactions are tested in wells coated with either TNF-α; negative control proteins IFN-γ, TNFR, His2B; or no protein. Anti-c-Myc antibody conjugated to HRP is used to detect binding. Data is average of two replicates and scale bar shows one times standard deviation above and below average.

We used ELISA binding experiments to confirm that the isolated antibody sequences encoded target-binding VH and VL domains. The germline families and the CDRs (complementarity determining regions) for these IgGs are shown in ESI Appendix Dataset S2.† We created scFv expression constructs by linking together paired VH- and VL-encoding DNA from fifteen individual cells obtained by sorting droplets positive for anti-TNF-α activity. We then PCR-amplified these linked chains using primers that add a T7 promoter upstream of the start codon and an in-frame myc epitope tag to 3′ end of the VL-gene (Fig. 3a and ESI Appendix Dataset S2†). We used the sequence of the anti-TNF-α antibody drug Adalimumab^33^ to create a positive control construct, and we created our negative control construct using paired VH- and VL-encoding genes from a rat B-cell making an irrelevant antibody (see Materials and methods for details). We used cell-free transcription and translation to produce the scFv proteins and we confirmed protein synthesis by dot blot. scFv #7 was generated from non-rearranged DNA and detectable protein was not synthesized. All the other scFvs were well expressed (Fig. 3b).

We added the cell-free-synthesized scFv proteins to wells coated with either TNF-α protein or one of three negative control proteins. We then detected binding using an anti-cMyc antibody. The positive control scFv bound strongly to TNF-α but not to mouse interferon (IFN-γ), recombinant TNF-receptor II (TNFR), Histone 2B (His2B), or to the no-antigen blank control well. As expected, virtually no signal was detected in wells loaded with a cell-free synthesis reaction lacking a DNA template. None of the sorting-derived scFv proteins showed significant binding to any of the negative control proteins. Twelve sorting-derived scFv proteins generated TNF-α binding signal that was roughly twice that of the negative control scFv or higher. Notably, scFv protein #12 generated significantly more anti-TNF-αsignal than the positive control scFv (Fig. 3c). These ELISA results indicate that the sequences isolated from droplet-sorted cells encode antibodies that bind to the target antigen TNF-α and do not cross-react with a panel of irrelevant proteins.

To demonstrate that our method significantly enriches for desired cells, and to identify relatively inefficient steps within the process, we report the success rate for each step of the process for two typical sorts performed with high and low sorting thresholds. The input is cultured rat splenocytes, of which ∼0.2% secrete anti-TNF-α antibody, as determined by fluorescent foci analysis.^34^ We generated TNF-α-binding scFv proteins from ∼5% and ∼10% of the droplets sorted in the low- and high-threshold sorts, respectively. We calculate the fold-enrichment by the overall process as the % of sorted cells converted into ELISA-positive scFvs/% of input cells that secreted anti-TNF-α antibody. The low- and high-threshold sorts delivered enrichments of ∼24-fold (∼5%/∼0.2%) and ∼50-fold (∼10%/∼0.2%), respectively (ESI Appendix Table S3†). This table also reveals that a major inefficiency in our process is the relatively low rate of antibody gene recovery from isolated single cells: ∼8% and ∼10% for the low- and high-threshold sorts, respectively. Straightforward modifications, such as improved primer design and efforts to reduce RNA degradation, *e.g.*, use of freshly cultured cells as input and reduced overall sorting duration, will improve gene recovery and will directly increase enrichment rates.

## Discussion

We have demonstrated a droplet-microfluidics platform to identify and verify correctly paired target-binding antibody genes from heterogeneous cell populations. We used a simple in-droplet bead-based binding assay and microfluidic droplet-sorting to isolate desired cells that secrete anti-TNF-α antibodies from a mixture of primary rat spleen cells containing a vast excess of irrelevant cells. Our droplet sorting devices included a passive droplet size-filter to remove merged droplets, which greatly reduced false positives and enabled isolation of cells that were rare (∼0.2%) in the frozen cell input. We recovered the antibody variable region sequences by single-cell RT-PCR, which maintains authentic heavy- and light-chain pairing, and we used cell-free protein synthesis and ELISA to characterize recombinant versions of the cell-secreted antibodies to confirm that the majority of the recovered heavy- and light–chain pairs encode antigen-specific antibodies. This platform requires no B cell immortalization or library preparation and provides an efficient means to mine human samples for medically relevant antibodies. Our system compartmentalizes individual cells in picoliter volumes to enable sensitive detection of secreted products, making it similar in principle to methods using microengraved wells.^21,22^ However, the droplet-based system may offer increased throughput by using rapid droplet sorting to isolate cells of interest. Our current platform lacks a mechanism to compensate for assay beads being out of focus during detection, and we cannot accurately quantify the antibody secreted and cannot determine binding affinities in-droplet. Potential solutions have been described, for example the use of two-color detection to normalize the antibody-binding fluorescence against bead calibration fluorescence,^8^ and a highly effective use of a magnetic field to align small detection beads in the focal plane.^30,35^ Further, with straightforward modifications, our platform can be used to isolate cells based on secretion of other proteins or chemicals, provided there is a means to generate a detectable signal within droplets.

## Materials and methods

We used droplet microfluidics techniques to perform in-droplet binding assays on antibody secreted from single cells. Microfluidic devices were used to sort droplets containing cells that secreted target-binding antibody.^7^ We dispensed the sorted droplets into wells of PCR strip-tubes at ∼0.3 droplets per well and performed single-cell reverse transcription PCR to recover the genes encoding the IgG heavy and light chains. We verified the VH- and VL-sequences by comparison to the IMGT database.^32^ We then used ELISA to confirm that these sequences encoded TNF-α-binding antibodies. To generate recombinant antibodies for binding tests, we used standard molecular biology techniques and Gibson Assembly to generate linear DNA constructs directing cell-free synthesis of scFv-format antibodies. Cell-free scFv production was confirmed by dot-blot and scFv binding was characterized by ELISA using plates coated with either TNF-α protein or with the negative control proteins mouse interferon-γ, recombinant human TNFRII, or recombinant Histone 2B (Active Motif 31492). The method conditions were optimized using mouse hybridoma cells, anti-TNF-α-secreting M357-101-4 cells (Sigma Aldrich 92030603) and anti-cMyc-secreting 9E10 cells (ATCC® CRL-1729™). New anti-TNF-α binding sequences were isolated using spleen cells from Sprague Dawley rats immunized with human TNF-α protein.

## Conflicts of interest

John Heyman, David Weitz, and Shaorong Chong have business interests in Spherebio. At the time we originally submitted this work, we were considering using some of the described methods for a commercial project to be performed by Spherebio. However, we decided against this project and currently have no plans to use the described method commercially.

## Supplementary Material

RA-010-D0RA04328A-s001

RA-010-D0RA04328A-s002

RA-010-D0RA04328A-s003

RA-010-D0RA04328A-s004

RA-010-D0RA04328A-s005
