# Supplementary material for: Rapid isolation of antigen-specific B-cells using droplet microfluidics
Source: RSC Adv. 2020 Jul 20;10(45):27006–13. doi: 10.1039/d0ra04328a (PMC9055518; doi:10.1039/d0ra04328a)
Supplement: RA-010-D0RA04328A-s001 [file RA-010-D0RA04328A-s001.pdf]

Electronic Supplementary Material (ESI) for RSC Advances.
This journal is © The Royal Society of Chemistry 2020

Supplementary Appendix for

High-throughput isolation of antigen-specific B-cells using droplet microfluidics

Ding, Ruihua; Hung, Kuo-Chan; Mitra, Anindita; Ung, W. Lloyd; Lightwood, Daniel; Tu, Ran; Starkie, Dale; Cai, Liheng; Mazutis, Linas; Chong, Shaorong; Weitz, David. A.*; Heyman, John A.*

***Corresponding Authors:**
John A. Heyman, 11 Oxford Street, Harvard University, Cambridge, MA 02138, USA.
Phone: 760-884-9961; Email: jheyman@g.harvard.edu

David A. Weitz, 29 Oxford Street, Harvard University, Cambridge, MA 02138, USA.
Phone: 617-496-2842; Email: weitz@seas.harvard.edu

This PDF file includes:

Supplementary Information Text: Materials and Methods
Figs. S1 to S4
Tables S1 to S3

Other supplementary materials for this manuscript include the following:

Dataset S1 (separate file): "Ding et al SI dataset 1 - IgG V region sequences"

Dataset S2 (separate file): "Ding et al SI dataset 2 - Germline allele information and CDR sequences for binding-tested IgGs"

Dataset S3 (separate file): "Ding et al SI dataset 3 - scFv Expression Constructs"

Supplementary Information Text:
Materials and Methods

Device fabrication

Devices are fabricated using photolithography, essentially according to published detailed methods (1); a brief summary is in the legend for **Fig. S1.** Briefly, masks with appropriate features are designed using AutoCad and printed onto transparent film by (CAD/Art Services, Inc. Bandon, OR). We spin-coat photoresist SU8-3025 (Microchem, Newton, MA, USA) onto silicon wafers (3-inch diameter, Type-P, 1S polished; University Wafer, S3P01SP) previously cleaned by compressed air. Photolithography masks are positioned onto the coated wafers and photoresist is cross-linked by UV exposure. We use propylene glycol monomethyl ether acetate (PGMEA; Sigma-Aldrich 537543) to remove the uncrosslinked photoresist. For all devices the height of the photoresist mold is about 25µm. The wafer is then treated with 0.2% 1H,1H,2H,2H-Perfluorododecyltrichlorosilane (Sigma Aldrich 448931) in HFE7100 (3M, USA) for 10-15 minutes. Polydimethylsiloxane (PDMS, Sylgard 184 Silicone Elastomer, Dow Corning, Midland, MI) is mixed 10:1 (w:w) with curing agent and degassed before pouring onto the mold. The device is further degassed and cured at 65°C for > 5 hours and then carefully peeled off the mold. We use biopsy punches (2mm diameter Harris Uni-Core, Ted Pella, Inc., Redding, CA) to create tubing holes to the channels. We use an isopropanol sonicating bath to remove punch-hole debris, remove excess isopropanol with compressed air, and then dry the PDMS slab at 65°C for > 30min. The PDMS slab is then bonded to a clean glass slide after careful cleaning and plasma activation of both surfaces. Sorting electrodes are fabricated into the device using low melting temperature solder (The Indium Corporation of America, wirebn-53307). Aquapel (PPG Industries, Pittsburgh, PA) is then flushed through channels to make channel surfaces hydrophobic.

Cell culture and staining

Anti-TNF-α-secreting M357-101-4 cells are purchased from Sigma Aldrich (92030603). Anti-cMyc-secreting 9E10 cells are from ATCC (ATCC® CRL-1729™). Hybridoma cells are cultured in complete DMEM media (Corning 10-013-CV supplemented with 10% FBS, Sigma part 2442; and 1% penstrep, Hyclone part SV30010). Cells were stained by adding 1 µl of CellTracker™ DeepRed (ThermoScientific C34565, reconstituted in dimethyl sulfoxide to a concentration of 1µg/µl) to 5ml of cells in culture and incubating overnight in 37°C cell incubator.

Bead preparation

Polystyrene beads with streptavidin coating (Spherotech SVP-60-5) are washed with PBS buffer then resuspended in PBS buffer with 85ng/µl biotinylated secondary antibody (Jackson 112-065-008 for anti-rat; Jackson 115-065-008 for anti-mouse) and rocked at 4°C overnight. The beads are washed 4 times with complete DMEM media immediately before encapsulation.

Bulk test of fluorescence concentration assay

Capture beads are resuspended in media with 40% Percoll (v/v, Sigma P4937). Typical bead concentration is 20 million per mL. Labeled antigen is present at 30ng/µl. Cells, if present, are at 20 million per mL. The mixture is incubated at 37°C and imaged after > 15min incubation or with continuous imaging.

Rat Spleen Cells

Rat spleen cells were isolated from Sprague Dawley rats immunized with human TNF-α protein. A single cell suspension of splenocytes was cultured using the method described by Tickle et al. (2015). Briefly, splenocytes at approximately 10,000 cells per ml in a 96-well plate were cultured using the mutant EL-4 feeder cell line system described by Zubler et al.(2). Cells were incubated statically for one week in a humidified environment at 37C with 5% CO_2, to allow for activation and expansion of the memory B cell

population. Following a week in culture the cells were harvested via centrifugation at 300xG, resuspended in 10% DMSO in fetal calf serum (FCS), aliquotted into cryovials and frozen in a controlled freezing container placed in a -80c freezer. These cryovials were then transferred to liquid nitrogen tank for storage. To determine the frequency of cells that secrete IgG- and antigen-specific IgG, we thaw an aliquot, remove dead cells using either Miltenyi dead cell removal kit (Miltenyi Biotec 130-090-101) or Lympholyte®-Rat density separation medium (Cedarlane CL5040) according to the manufacturer's instructions, and use the fluorescent foci method (Tickle et al., 2015) to characterize the cells. Prior to use in microfluidic experiments, frozen primary cell stocks are thawed at 37°C and added to 10ml pre-warmed complete DMEM. The cells are pelleted for 5min at 400g and resuspended in media. Resuspended primary cells and cultured hybridoma cells are passed through a 40µm nylon strainer (VWR 10199-654). We add 0.25 µl DNAseI (New England Biolabs N0303) and 1.2 µl 1M $MgCl_2$ per 0.5ml cell solution and incubate at 37°C for 15min. Cells are then pelleted, resuspended in complete DMEM and purified. Then the cells are chilled on ice for >10min to prevent antibody secretion prior to encapsulation into droplets.

Droplet making

Cells and beads are resuspended in separate media solutions containing 40% (v/v) Percoll (Sigma P4937), 30ng/µl labeled antigen (~1.7 µM) and 2% (v/v) RNAse inhibitor (RNAsin® Plus Promega N2611). We use HFE7500 (3M, USA) containing 2% (w/v) surfactant (008-FluoroSurfactant, Ran Biotechnologies, USA) as the continuous phase. Harvard PHD2000/2200 pumps (Harvard Apparatus, Holliston, MA) are used for liquid injection. Typical flow speed is 150µl/hr for each of the aqueous solutions and 700µl/hr for the oil phase. Typically, we have an average of 3-5 capture beads per droplet and one cell per 3-5 droplets, so that most droplets have at least one capture bead and few droplets have more than one cell. We track droplet production by monitoring the fluorescence downstream of the droplet-forming nozzle via PMT. Droplets are collected into 1mL syringes (BD Luer-Lok™ 309628) and moved to 37℃ cell culture incubator for desired period, typically 2 hours. We assume that cells will secrete at different rates, ranging from ~4 to ~10,000 molecules per second (3). In a droplet containing a cell that secretes at this maximum rate, secreted antibody will reach 1.7 µM after 1.4 hr incubation at 37 °C. Because cells do not secrete at their maximum rate immediately upon shift from 4 °C to 37 °C, and because most cells will have maximum secretion rate <10,000 antibody molecules per second, we believe that in almost all droplets the labeled antigen (alexa-488 labeled TNF-α protein, 1.7 µM) will be in excess of cell-secreted antibody. However, this high concentration of fluorescent antigen also reduces detection sensitivity, which is related to the ratio of bead-bound to unbound fluorescent antigen.

Droplet sorting

After droplet incubation, we use PEEK tubing (Western Analytical Products, Tub PEEK Nat Tubing Sleeve PEEK Natural 1/32" OD x .008" ID) to attach the droplet-collection syringe to a sorting device. HFE7500 with 0.2% surfactant is used as the oil in the filter and downstream spacing. The sorting device (Fig. S2) can be operated at speeds over 100 Hz; however, sorting at the maximum rates may result in increased collection of undesired cells. Thus, when performing longer sorting experiments to isolate rare events, we use a flow rate of 15µl/hr for droplets, 200µl/hr for filter oil and 250µl/hr for spacing oil, and droplets pass through the sorting junction at ~70Hz. We use the "observation trap" on the microfluidic device (Fig. S2, element j) to tune and verify sorting thresholds. We use our sorting instrument to analyze a fraction of input droplets and generate a scatterplot of droplet fluorescence intensity. All droplets contain the same concentration of antigen. For example, in Figure 2E, the vast majority of droplets are in the yellow region, having a droplet peak intensity of ~0.5 and droplet average intensity of ~0.45. Anything with droplet average intensity significantly higher than ~0.5 is assumed to be an abnormal droplet, e.g., shrunken during incubation or containing very high fluorescence due to particulate fluorescent antigen. Droplets lacking a fluorescent bead will have pedestal-shaped peaks where the droplet peak intensity is essentially the same as the droplet average intensity. Only the desired droplets have a droplet peak

intensity significantly higher than the droplet average intensity. Thus, we sort based on droplet peak intensity relative to droplet average intensity. The droplet peak intensity may be from a single bright bead, or the cumulative signal of several beads detected simultaneously. In practice, we select a sorting threshold in two steps. We monitor droplets, usually >20,000, and select a preliminary sorting threshold based on the signal distribution. We then perform a limited sort and collect a small number of droplets into the observation trap. We then disconnect the device and image the trap at a confocal microscope to confirm sorting. We then re-connect the device and plug the trap outlet (Fig.S2, element k) to direct sorted droplets through the collection outlet (Fig.S2, element i). After the flows equilibrate, we sort into a collection tube. Use of the observation trap greatly improves sorting accuracy, though overall sorting rate is reduced. In a typical sort, which included several breaks to fine-tune sorting thresholds, we screened ~600,000 droplets in ~5hr, giving a rough average speed of 30Hz. ~33% of the droplets contained a cell and at least one bead, giving an overall rate of ~10 cells per second. To enable use of a pipetman to dispense sorted droplets, we collect sorted droplets into a ~20µL emulsion of 50 µm diameter "dilution" droplets generated by droplet microfluidics and consisting of 75.5% water, 20% Percoll, 2.5% PBS and 2% (v/v) RNAse inhibitor (Promega N2611). Droplets are then gently mixed and dispensed by pipetting into PCR tubes.

Identification of sorted hybridoma cells

Analysis of sorted droplets: We perform reverse transcription and nested PCR (RT-nPCR) on sorted droplets to identify the encapsulated cells as either anti-cMyc- or anti-TNF-alpha- hybridoma cells. Primer sequences are given in (*SI Appendix* Table S2). Prior to performing RT-nPCR on sorted droplets, we demonstrate the sensitivity and specificity of the assay through analysis of samples containing anti-cMyc- and anti-TNF-α- hybridoma cell mRNA mixed at equal ratios (mRNA concentration is expressed in "cell equivalents"). We assemble on ice reverse transcription reactions containing 0.5µM "mouse IgG Heavy chain RT" reverse primer, 0.25µM "TSO-mouse" template switching oligo, 0.5ul Superscript™ IV reverse transcriptase, 4µl 5x Superscript™ IV reverse transcription buffer, 1µl RNAsin® Plus, 5 mM DTT, the indicated number of cell equivalents as template, and dh20 to final volume of 20µl. To ensure efficient template-switching, we perform the RT in two steps. Initially, we incubate for ten minutes at 55°C, then lower the temperature to 42°C, add 0.5µl MuLV enzyme (NEB M0253S) and incubate 40 min at 42°C, then ramp to 4°C. Samples are frozen at -20°C or used immediately for PCR. One microliter of each RT mixture is used for 20µl initial PCRs (iPCR) containing: 0.25µM forward primer "O-LA1" (anneals to the sequence added during template switching), 0.25µM reverse primer "Mouse IgG 1st PCR" (anneals perfectly to both the anti-cMyc and anti-TNF-α heavy chain-encoding cDNA), 0.4µl Taq polymerase (ThermoFisher EP0401), 2µl 10x $(NH_4)_2SO_4$ PCR buffer, 2 mM $MgCl_2$, 0.2 mM dNTPs (each), and dh20 to 20µl total volume. The samples are cycled using a touchdown protocol: Initial denaturation at 94°C for 3 min; then 9 cycles of: 94°for 30 sec, anneal for 30 sec using temperature decreasing by 1°C per cycle (62°C to 54°C), extend 1 min 72°C; then 21 cycles of 94°C for 30 sec, anneal at 54°C for 30 sec, and extend at 72°C for 1 min; then a final extension at 72°C for 5 min, followed by a hold at 4°C. A fraction, 2.5%, of each iPCR is then used as template in 10ul nested PCRs (nPCR) containing a primer pair specific for anti-cMyc heavy chain cDNA (antiMyc Forward, antiMyc Reverse, 0.25µM each) and a pair specific for anti-TNF-α heavy chain cDNA (antiTNFforward, antiTNFreverse, 0.25µM each), 0.2µl Taq polymerase (ThermoFisher EP0401), 1µl 10x $(NH_4)_2SO_4$ PCR buffer, 2 mM $MgCl_2$, 0.2 mM dNTPs (each), and dh20 to 10µl total volume. The cycling protocol uses an initial denaturation at 94°C for 3 min; then 30 cycles of 94°C for 30 sec, anneal at 54°C for 30 sec, and extend at 72°C for 1 min; then a final extension at 72°C for 5 min, then samples are held at 4°C. The nPCR samples were separated on 3% agarose gel, then visualized by ethidium bromide staining and UV illumination.

To analyze sorting, reverse transcription is performed essentially as described above, but in small volumes to allow use of the entire RT reaction product as template for iPCR. We perform reverse transcription in sets of 8-well PCR strips. We prepare 100µl of reverse transcription cocktail comprising 0.5µM mouse IgG Heavy chain RT reverse transcription primer, 0.25µM TSO-mouse template switching oligo, 0.5 mM each dNTP, 5µl RNAsin® Plus RNAase inhibitor, 20µl 5x Superscript™ IV reverse transcription buffer, 5 mM DTT, 2.5µl Superscript™ IV reverse transcriptase, 2.5µl MuLV reverse transcriptase, 1 mM $MnCl_2$, and dh20 to final volume of 100µl. We add 1µl cocktail to each well of dispensed droplets (see "Droplet sorting" section above for droplet dispensing details) and place the strips at -80C for >80 min to freeze-break the droplets. We transfer the strips to a thermocycler, incubate ten minutes at 55°C, then lower the temperature to 42°C and incubate for 40 minutes, and either freeze the samples at -20C or place on ice for immediate use in iPCR. The iPCR master mix contains 0.25µM O-LA1 forward primer, 0.25µM Mouse IgG 1st PCR reverse primer, 9µl Taq polymerase, 45µl 10x $(NH_4)_2SO_4$ PCR buffer, 2 mM $MgCl_2$, 0.2 mM dNTPs (each), and dh20 to 360µl total volume. We add 4ul of this mix directly to the reverse transcription reaction wells and cycle using the touchdown protocol detailed above. The nPCR master mix contains primers specific for the anti-cMyc heavy chain chain (antiMyc Forward, antiMyc Reverse, 0.25µM each) and primers specific for the anti-TNF-α heavy chain (antiTNFforward, antiTNFreverse, 0.25µM each), 6.5µl Taq polymerase, 45µl 10x $(NH_4)_2SO_4$ PCR buffer, 2.5 mM $MgCl_2$, 0.16 mM dNTPs (each), and dh20 to 360µl total volume. We aliquot 4µl nPCR master mix into PCR tubes, then use a multi-channel pipette to transfer 1µl of each iPCR to wells containing the nPCR mix. We cycle using the above nPCR conditions. The PCR products are electrophoresed on 3% agarose gel and bands are visualized by ethidium bromide staining and UV illumination. For some PCR analyses, we multiplexed the nested PCR by combining the anti-cMyc and anti-TNF-α primer sets in each reaction. Because one of the anti-cMyc primers can bind with mismatches to DNA encoding the anti-TNF-α heavy-chain, multiplex reactions performed on samples containing anti-TNF-α cells often generated a 300bp product in addition to the authentic 166 bp product. When this occurred, we re-tested the samples by performing the nested PCR in a non-multiplex fashion.

Gene recovery from sorted Rat splenocytes

Reverse transcription was performed in a modified Superscript™IV reaction (ThermoFisher 18090050). Briefly, ~0.5µl sort-emulsion droplets are added to ~3.8µl reaction mixture consisting of 6.64 units RNAsin, 10nmol dNTP, 50nmol dCTP, 0.4pmol Rat IgG Heavy RT primer, 0.1pmol Rat IgG Light RT primer, and 0.1pmol Rat B2M primer (*SI Appendix* Table S2). The mixture is frozen at -80°C for > 40min, then heated to 72°C for 3min and immediately incubated on ice for more than 1 min. These steps break the droplets and allow primer annealing. We next add 5.7µl of solution containing 10µmol Betaine, 2µl Superscript™IV buffer, 0.25µl Superscript™IV enzyme, 50nmol DTT, 60µmol $MgCl_2$ and 20pmol TSO DNA is added to each tube, mixed, and spun down. Reverse transcription is carried out at 37°C for 30min, 4°C for 45min then 37°C for another 30min, finally the enzyme is deactivated at 80°C for 10min. We PCR-amplify VH and VL DNA using Platinum™ SuperFi™ (Thermo Fisher 12358010) according to manufacturer's specifications. When amplifying cDNA from template-switching RT reactions, we use O-LA1 and I-LA1 as the forward primers for the initial and nested PCR, respectively. We use RatHeavyFirst and RatHeavyNest primers and RatLightFirst and RatLightNest primers (*SI Appendix* Table S2) for first round and nested PCR of V heavy and V light genes, respectively. Alternatively, we perform PCR using primer mixtures provided by UCB Pharma for initial and nested PCR(4). DNA is gel purified (Sigma NA1111-1KT) followed by Zero-blunt TOPO™ cloning (ThermoFisher K283520). Transformed colonies are incubated overnight at 37°C on agar plates with appropriate antibiotic (Ampicillin or Kanamycin). At least 4 colonies from each transformation are picked and plated onto stock plates. We identify clones that contain plasmids with VH- and VL-encoding inserts by performing colony PCR using M13 forward and reverse sequence primers, and then we purify (QIAquick PCR cleanup kit, part 28106) and sequence the VH- and VL- products using Sanger sequencing with primers annealing to the T7 and T7 terminator sequences. We compare the sequences

with the IMGT database to confirm inserts encode VH- and VL- domains. Although we occasionally isolated more than one distinct putative heavy- or light-chain sequence from a single well, we used only one heavy-chain and one light-chain to create that well's scFv expression construct.

Cell-free synthesis of single-chain antibody

We PCR-amplified the IMGT-verified VH- and VL- sequences using forward primers that add pCOAT-DB(5) sequence to the 5' end of the VH DNA (pc-VH Forward primer) and linker-encoding sequence to the 5' end of the VL DNA (pc-VL Forward primer); the reverse primers add linker-encoding sequence to the 3' end of the VH DNA (pc-VH reverse primer) and pCOAT-DB sequence to the 3' end of the VL DNA (pc-VL reverse primer) (*SI Appendix* Table S2). We gel-purify these PCR products and combine paired VH- and VL- amplicons with a double-stranded linker DNA and linearized pCOAT-DB vector in NEBuilder HiFi Assembly reaction mixture (E2621) to generate circular plasmids. The sequence for the positive control is derived from adalimumab (CAS registry number 331731-18-1, sequence available at https://www.drugbank.ca/drugs/DB00051). We transform these reactions into competent *E. coli* and identify insert-containing clones by colony-PCR using primers flanking the pCOAT-DB insertion site. Clones with full-length insert are cultured overnight in ~5ml media with antibiotics. DNA is isolated from these cultures by Miniprep (Qiagen 27106) and further verified by Sanger sequencing. To minimize the effect of PCR-induced mutagenesis, we tolerate one amino acid difference between the pCOAT-DB inserts and the original IMGT-verified VH and VL sequences. The double-stranded linker DNA encodes the sequence GGGGSGGGGSGGGGS to link heavy and light chain domains (6). We perform sequential PCR amplifications using "first mp primers" and then "second mp primers" to generate linear cell-free protein synthesis constructs from these sequence-verified plasmids. These reactions add a T7 promotor and DNA encoding a myc epitope tag and transcription terminator to the 5' and 3' ends of each amplicon, respectively. The presence of the myc epitope in synthesized scFv molecules enables use of HRP-conjugated anti-myc antibody as the detection reagent in ELISA and dot blot experiments. The product is verified using gel-electrophoresis and gel-purified (Qiagen 27106). The concentration is measured with spectrophotometer (NanoDrop Technologies, Inc. ND-1000). We produce scFv proteins by using 300ng each PCR product in 25µl cell-free protein synthesis reactions (PURExpress® protein synthesis kit, New England Biolabs E6800). The mixtures are incubated at 37°C for 4hr and then stored at 4˚C ice. Although the PURExpress® buffer includes the reducing reagent DTT, protein synthesis in these conditions generates target-binding scFvs, as shown by target-specific binding of the positive control, an scFv derived from adalimumab. Further, we made power law correlations using DTT half-life data (provided by Sigma - https://www.sigmaaldrich.com/content/dam/sigma-aldrich/docs/Sigma/Product_Information_Sheet/d9163pis.pdf) to estimate a 30 minute half-life in our cell-free synthesis conditions. Thus, at the start of the third hour of incubation, >95% of the input DTT should be degraded and not provide reducing activity.

scFv ELISA

We use ELISA to test binding of cell-free synthesized scFv proteins to the proteins TNF-α (from UCB), mouse Interferon-γ (BioLegend 575306), recombinant human TNFRII (Peprotech 310-12) and recombinant Histone 2B (Active Motif 31492). All are dissolved at 8ng/µl in PBS (Corning 21-040-CV). We add 47µl antigen solution to bottom of each well (Universal Binding plate, Thermo 9502227) and incubate at room temperature for >3 hours. We then wash 3 times with wash buffer consisting of PBS with 0.1% BSA (Sigma A7906) and 0.05% Tween 20 (Sigma P9416) and add 400µl blocking buffer (wash buffer containing 5% (w/v) powdered non-fat milk) to each well and block overnight at 4˚C. We then wash wells 3 times with wash buffer and add 4µl cell-free protein synthesis mixture diluted in 50µl wash buffer to each well. We rock the plate at room temperature for >45min, then wash 3 times with 180µl wash buffer and then add 100µl HRP-conjugated rabbit anti-myc antibody (Genscript A00173) at 0.1ng/µl in wash buffer. We incubate at room temperature with rocking for >30min, then wash 5 times using wash buffer,

and then add 100µl HRP substrate (Biolegend 421501) to each well. We allow the reaction to proceed for several minutes at room temperature and stop by adding 100µl stop solution (Biolegend 77316). We determine the colorimetric signal intensity with a Synergy H1m plate reader (BioTek).

Dot Blot

Cell-free synthesis of scFv proteins was confirmed by dot blot. We pipetted 1µl of each cell-free synthesis reaction onto nitrocellulose membrane and air-dried for >30min. “Int. Ctrl.” = internal control, a sample known to be detected by anti-c-Myc dot blot. The membrane was incubated at 4˚C overnight and washed using wash buffer. The membrane was then covered in HRP-conjugated mouse anti-myc antibody (Genescript A00863 diluted to 0.1ng/µl in wash buffer), rocked at room temperature for >30min and washed wash buffer (three washes, each at least 15min). PierceTM ECL substrate (Thermo 32109) was used for chemiluminescence signal generation and membrane was imaged using an AlphaInnotech ChemiImager (ProteinSimple).

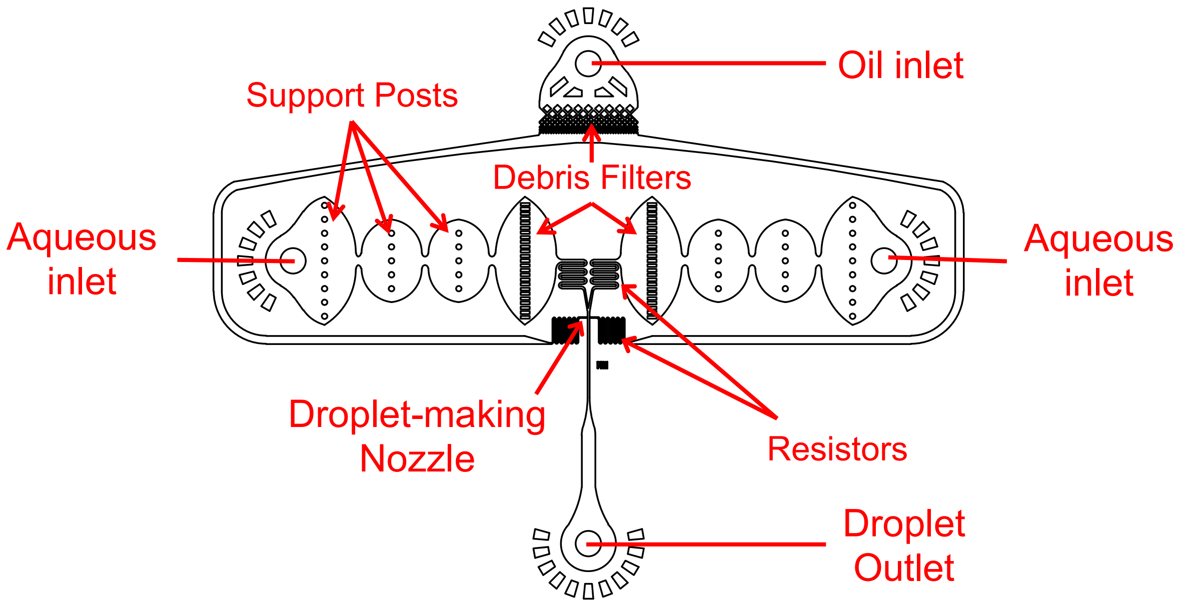

Figure S1. Dropmaker design. Solutions containing cells and/or beads in cell culture medium are injected from either of the aqueous inlets. The hourglass shape of the channels helps to disrupt aggregated beads and cells. The PDMS posts prevent the PDMS channels from collapsing. The PDMS filters in the final aqueous hourglass chamber and in the oil inlet remove debris that may clog channels. The serpentine-shaped channel regions are resisters that help maintain consistent flow rates. The two aqueous solutions join immediately upstream of the dropmaking nozzle.

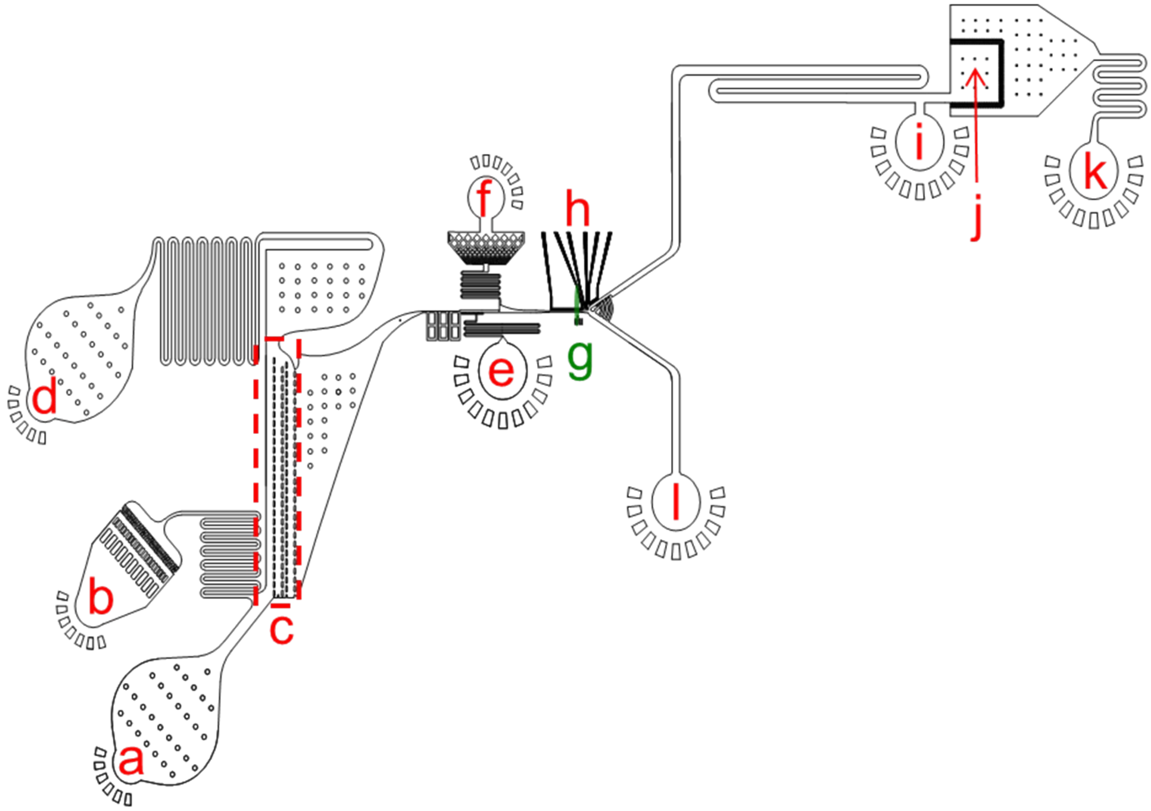

Figure S2. Dropsorter design. Droplets are injected from droplet inlet (a) and spaced by oil injected into inlet (b). Abnormally large droplets are excluded by droplet filter (c) and exit the device from merged droplet outlet (d). Excess oil between irregularly spaced droplets is removed through a filter upstream of device outlet (e). Oil is added from oil inlet (f) to uniformly space the droplets. This packing and uniform re-spacing of droplets improves sorting speed and accuracy. After spacing, droplets are exposed to slit-laser excitation and the resulting fluorescence is detected (g). To sort droplets, activated electrodes (h) generate a temporary electrical field and steer desired droplets to the upper channel. The droplets can then be collected for optical observation by plugging the collection outlet (i) and imaging the observation trap (j). Alternatively, the trap outlet (k) can be plugged and sorted droplets are collected from collection outlet (i). Discarded droplets exit the device through the waste channel (l).

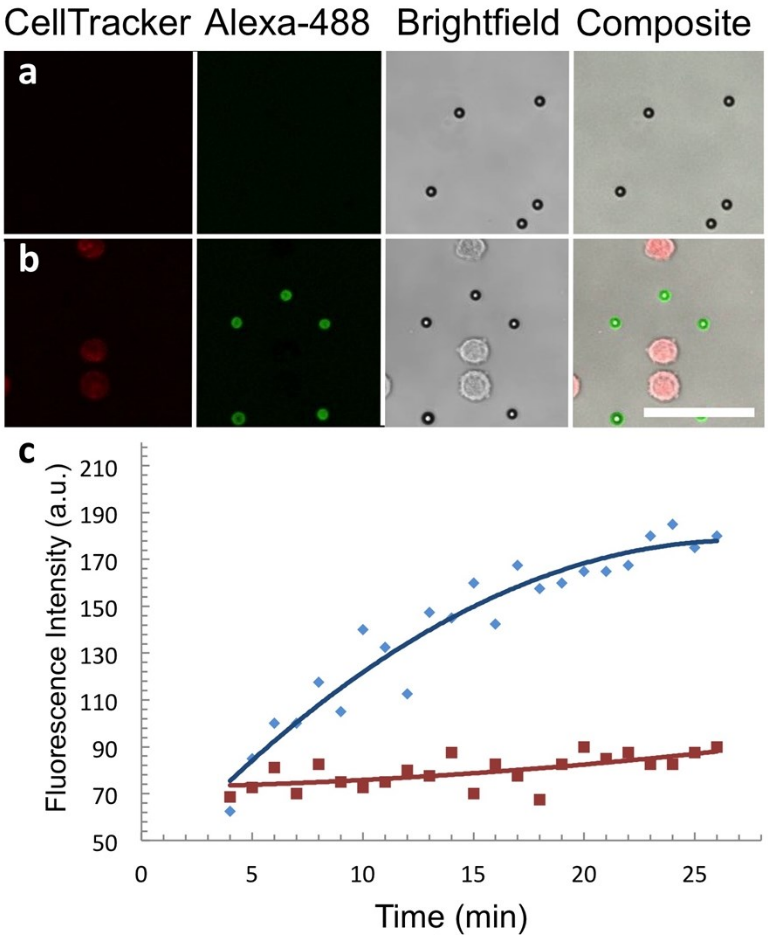

Figure S3. Binding assay in bulk. Anti-mouse-antibody-coated beads and fluorescently labeled TNF-α protein are incubated without (a) or with (b) anti-TNF-α-secreting hybridoma cells. Images are taken 6 hours after reagents are mixed. (c) Capture beads and fluorescent TNF-α incubated in the absence or presence of anti-TNF-α secreting hybridoma cells. Bead fluorescence intensity was measured at indicated time points over 30 minutes. Each data point is the average intensity of 4 beads measured using confocal microscopy. Scale bars: 75µm.

Cell type in reverse transcription (RT) reaction	NA	Anti-cMyc hybridoma			Anti-TNF-α hybridoma			NA
Cell Equivalents in reverse transcription	0	1	20	1000	1	20	1000	NA
% of the RT reaction used as iPCR template	5	5	5	5	5	5	5	NA
% iPCR used as nPCR template	2.5	2.5	2.5	2.5	2.5	2.5	2.5	NA

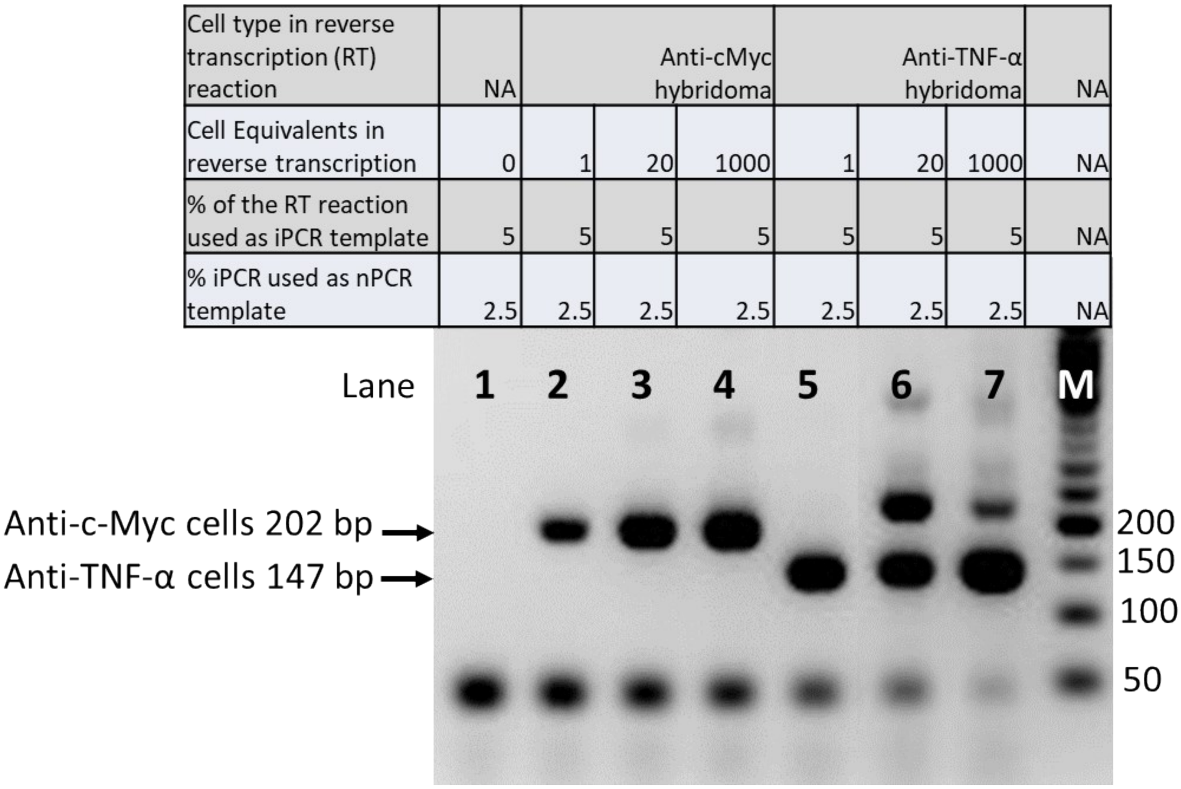

Figure S4. Sensitivity and specificity of hybridoma RT-PCR. mRNA was purified from counted anti-cMyc and anti-TNF-α hybridoma cells. The mRNA was combined to form a sample containing one thousand cell equivalents of each cell type per microliter. Reverse transcription was performed in 20µl reactions using the indicated number of cell equivalents as template. One microliter of each RT mixture (5% of the total RT reaction) was used for initial PCR (iPCR) using the template-switching forward primer O-LA1 and the reverse primer Mouse IgG 1st PCR, which anneals perfectly to both the anti-cMyc and anti-TNF-α heavy chain cDNA. A fraction, 2.5%, of each iPCR was then used as template in 10ul nested PCRs (nPCR) containing a primer pair specific for anti-cMyc heavy chain cDNA and a pair specific for anti-TNF-α heavy chain cDNA. The marker lane, M, was loaded with 50bp ladder. The predicted band size for each nPCR primer pair is indicated at left. Marker band sizes are indicated in basepairs at right.

Table S1. Comparison of antibody discovery methods.

Method and name, with citations	Description	Antibody format during screening	Instru-mentat-tion require-ment	Through-put	Comments
Hybridoma generation and selection (7-9)	Animal spleen cells are immortalized by fusion to myeloma cells. Clones are isolated by sequential rounds of expansion and selection.	Full-length IgG	Low	Low	Some loss of antibody repertoire during cell fusion. Requires repeated rounds of expansion and selection. Hundreds of clones are screened. Accelerated commercial services (10) require ~40 days from cell fusion step to completion of hybridoma subcloning.
Display methods using randomly paired heavy and light chains (11-15)	IgG heavy- and light-chain genes are isolated from cells from naïve or immunized animals and assembled into DNA constructs to display the single-chain format molecules (scFv), typically on phage or yeast cells.	Typically as scFvs	Low	High	Library construction and screening can be accomplished in 2-4 weeks (15). Mispairing of heavy- and light-chain genes can result in low-affinity antibodies that may require affinity maturation. (16)
Display methods pairing heavy and light chains in droplets (17-19)	Single-cell reverse transcription and linking PCR are performed in droplets to maintain correct pairing of IgG heavy- and light-chain genes. Display and panning are performed as in standard display methods.	Typically as scFvs	Medium-low	High	Initial publications demonstrate isolation of high-affinity antibodies with no affinity maturation required. Droplet-making equipment is inexpensive and simple to use. Library construction requires molecular biology expertise.
Peptide barcoding ("Nestlink") of potential binders (20)	Peptide barcodes ("Flycodes") are genetically fused to the potential binders and next-gen sequencing assigns each flycode to a potential binder. Library is expressed as a pool of barcoded proteins and binders are isolated. Flycodes are then identified by liquid chromatography-tandem mass spectrometry (LC-MS/MS).	nanobody	High	Medium-high	LC-MS/MS expertise is required. Library construction is time-consuming. Demonstrated uses include isolation of Camelid antibodies.
Microwell-based (21-23)	High-density microwell arrays are used to perform binding assays on antibody secreted by single cells. Cells secreting binding antibody are isolated and paired heavy- and light-chain genes are retrieved.	Secreted full-length IgG	High	Medium-High	Wells can be imaged over time. Efficient cell retrieval from wells requires instrumentation.
Glass capillary microarrays "uSCALE" (Single Cell Analysis and Laser Extraction) (24, 25)	Glass capillary microarrays are used to perform binding assays on antibody secreted by single cells. Intact cells that secrete binding antibody are ejected from wells and paired heavy- and light-chain genes are isolated by single-cell RT-PCR.	Secreted full-length IgG	High	High	Requires highly specialized instrumentation.
FRET-based in-droplet binding assays (26)	FRET-based binding assays and performed in microfluidic droplets. Droplets with binding activity are sorted; paired heavy- and light-chain genes are isolated from single cells	Secreted full-length IgG	Medium-high	High	FRET assays read-out avoids problems associated with loading assay beads into droplets, but FRET assays may be difficult to develop for some antibody-target pairs.

Method and name, with citations	Description	Antibody format during screening	Instru-mentat-tion require-ment	Through-put	Comments
Gel Encapsulated Microenvironment (GEM) assay (27)	Cells are embedded in hydrogel droplets with reporter beads. Antibody secretion and specificity are tested using fluorescence-concentration assay and desired cells are manually picked for sequencing	Secreted full-length IgG	Medium-high	Tens of millions of splenocytes per test	Gel particles are large and can contain more than one cell. Gel particles can be isolated at rates of >200/hr by skilled researcher.
Nanopens (28)	Cells are loaded into nanopens using OptoElectro Positioning and gravity. The cells secreting desired antibodies are identified with bead-based assay. Cells are then harvested using OptoElectro manipulation.	Secreted full-length IgG	Medium-high	Several thousand pens per chip	Requires complicated and precisely fabricated chips and specialized instruments for cell loading and imaging.
High-throughput sequencing of the variable regions of B cells, often referred to as Immunoglobulin Repertoire sequencing (29)	Next-Gen sequencing is used to determine total immune repertoires and bioinformatics is applied to identify, e.g., clones that expand in response to exposure or immunization. Ig-seq is very powerful when combined with functional data: total repertoires can be bioinformatically compared with sequences identified through functional screening of repertoire subsets to identify potential binders with improved features.	Study-dependent	Low to medium	High	Does not suffer from biases due to cell immortalization. Requires bioinformatics expertise. Single-cell platforms such as nanowells or droplet-based barcoding are required for true heavy- and light- chain pairing.
Nanometer-sized, magnetically-aligned capture-bead-in-droplet assays described in Gerard et al (30)	Fluorescence-concentration assays are performed in microfluidic droplets using ~1300 magnetic capture beads/droplet. Assay determines amount of antibody secreted and amount bound, enabling sorting based on affinity. After droplet sorting, cells are released from droplets and re-encapsulated for single-cell barcoding sequencing to identify correctly paired heavy- and light-chain genes.	Secreted full-length IgG	Medium-high	High	The use of nanometer-sized magnetically alignable capture beads improves signal-to-noise and reduces chances of beads clogging channels but adds some complexity to device fabrication. Barcode-based identification of V-region sequences requires technical and bioinformatics expertise, and recombinant constructs must be synthetically generated.
Micron-sized capture-bead-in-droplet assays (this study)	Fluorescence-concentration assays are performed in microfluidic droplets to identify cells that secrete target-binding antibody. This system, as currently described, does not allow for affinity measurements. Droplets in which cell-secreted antibody binds target protein are sorted and dispensed into individual wells. Single-well RT-PCR is used to isolate paired heavy- and light-chain V-genes from single droplet-sorted cells.	Secreted full-length IgG	Medium	Medium-high	Bead-based concentration assay is easily modified for different protein targets. Some droplets lack a detection bead, resulting in some inefficiency. Unlike Gerard et al., (30), DNA to generate recombinant clones is isolated directly from individual sorted cells, which may be more efficient and economical for DNA sequencing and antibody reconstruction, especially when desired cells are rare. Second encapsulation is not required, which can be advantageous if cells are damaged and likely to leak mRNA during barcoding encapsulation.

Table S2. DNA primers. K=T or G; /SP18//SP18 = two tandem 18-atom hexa-ethyleneglycolspacers; rGrGrG= three RNA G bases; R = A or G; S = G or C; /5Biosg/ = 5' Biotin; +G = Locked Nucleic Acid base G.

mouse IgG Heavy chain RT	CAGGGATCCAKAGTTC
TSO-mouse	/SP18//SP18/AAGCAGTGGTAACAACGCAGAGTACGCrGrGrG
Mouse IgG 1st PCR	AGGGAAATARCCCTTGACCAG
O-LA1	CAGTGGTAACAACGCAGAG
I-LA1	GGTAACAACGCAGAGTACG
antiMyc Forward	CTGGAGGGTCCCTGAAACTC
antiMyc Reverse	AGGTACAGGGCGTTCTTGTC
antiTNF forward	GCTTGAGTGGGTTGCTGAAG
antiTNF reverse	GTGTCTTCAGCTCCTAAGTTGTTC
Rat IgG Heavy RT	CCACMACACASGTGACC
Rat IgG LightRT	CACTTGACACTGATGTCTCTGG
Rat B2M RT	ACGGTTTTGGGCTCCTTCAGAG
TSO-rat	/5Biosg/AAGCAGTGGTAACAACGCAGAGTACGCrGrG+G
RatHeavyFirst	GGGAAGATGAAGACAGATG
RatHeavyNest	GACAGGGCTCCAGAGTTCC
RatLightFirst	CTGTACGTGCTGTCTTTGC
RatLightNest	CACTTGACACTGATGTCTCTGG
pc-VH Forward	GGTGGTCTCGAGGGTGGTGGTTCT-<~25bp gene specific sequence>
pc-VH Reverse	CTCCACCACCGCTACCTCCGCCGCC-<~25bp gene specific sequence>
pc-VL Forward	GGAGGAAGCGGAGGAGGCGGGTCC-<~25bp gene specific sequence>
pc-VL Reverse	TAATTAAAGGCCTCCTGCAGGATTA-<~25bp gene specific sequence>
Linker sequence	GGCGGCGGAGGTAGCGGTGGTGGAGGAAGCGGAGGAGGCGGGTCC
First mp forward	GGGCTTAAGTAAAGGAGGAAAAAATATGAGTAACAAAAAAACAACAGCAGGTG
First mp reverse	CCTCTTCGGAAATCAACTTTTGCTCGCTACCTCCACC - <~25bp gene specific sequence>
Second mp forward	GCGAATTAATACGACTCACTATAGGGCTTAAGTAAAGGAGG
Second mp reverse	AAACCCCTCCGTTTAGAGAGGGGTTATGCTAGTTACAAGTCCTCTTCGGAAATCAAC

Sorting Threshold	# Droplets observed	# Droplets sorted	Heavy- and light- chain DNA recovered	Heavy- or light- chain DNA recovered	scFv constructs made and ELISA- tested	TNF-α-binding scFvs[b]	% of sorted cells converted into positive ELISA scFvs	Fold enrichment by the overall process[c]
Low	320,000	117	10 (10/117=~8%)	14	9	6	~5% (6/117)	25 (~5%/~0.2%)
High	380,000	38	4 (4/38=~10%)	11	4	4	~10% (4/38)	50 (~10%/~0.2%)

Table S3: Summary of separate low- and high- threshold sorting experiments. We generated droplets containing cultured rat splenocytes[a] and anti-TNF-α fluorescence detection reagents, incubated to allow antibody secretion, and performed sorting using low and high fluorescent signal thresholds. In the low threshold sort, ~0.04% of the observed droplets had an above-threshold fluorescent signal, resulting in electrode activation to sort 117/320,000 observed droplets. We recovered both the heavy- and the light-chain-encoding DNA from fourteen of these droplets and used cell-free synthesis to make scFv constructs from nine of the recovered heavy- and light- chain pairs. Six of these scFv proteins clearly bound to TNF-α[b]. In the high threshold sort, ~0.01% of the observed droplets had an above-threshold fluorescent signal, resulting in electrode activation to sort 38/380,000 observed droplets. We recovered both the heavy- and the light- chain-encoding DNA from four of these droplets and used cell-free synthesis to make the corresponding four scFv proteins. All four scFv proteins clearly bound TNF-α[b]. The low- and high-threshold sorts showed ~25-fold and ~50-fold enrichment, as judged by the % of sorted cells converted into ELISA positive scFv/the % positive cells in the input. Superscript meanings - **a**: Cells secreting anti-TNF-α antibody comprised ~0.2% of the input cell population, as determined by fluorescent foci analysis (Clargo, 2014, mAbs). **b**: Produced a signal in TNF-α ELISA that was >175% the signal generated by a negative control scFv. **c**: The fold enrichment by the overall process is the % sorted cells converted into ELISA-positive scFvs / % of input cells that secreted anti-TNF-α antibody.

References used in Supplementary Information Appendix:

1. Mazutis L*, et al.* (2013) Single-cell analysis and sorting using droplet-based microfluidics. *Nat Protoc* 8(5):870-891.
2. Zubler RH*, et al.* (1985) Mutant EL-4 thymoma cells polyclonally activate murine and human B cells via direct cell interaction. *J Immunol* 134(6):3662-3668.
3. Eyer K*, et al.* (2017) Single-cell deep phenotyping of IgG-secreting cells for high-resolution immune monitoring. *Nat Biotechnol* 35(10):977-982.
4. Clargo AM*, et al.* (2014) The rapid generation of recombinant functional monoclonal antibodies from individual, antigen-specific bone marrow-derived plasma cells isolated using a novel fluorescence-based method. *MAbs* 6(1):143-159.
5. Zhou Y*, et al.* (2014) Engineering bacterial transcription regulation to create a synthetic in vitro two-hybrid system for protein interaction assays. *J Am Chem Soc* 136(40):14031-14038.
6. Huston JS*, et al.* (1988) Protein engineering of antibody binding sites: recovery of specific activity in an anti-digoxin single-chain Fv analogue produced in Escherichia coli. *Proc Natl Acad Sci U S A* 85(16):5879-5883.
7. Kohler G & Milstein C (1975) Continuous cultures of fused cells secreting antibody of predefined specificity. *Nature* 256(5517):495-497.
8. Greenfield EA (2014) *Antibodies : a laboratory manual* (Cold Spring Harbor Laboratory Press, Cold Spring Harbor, New York) Second edition. Ed pp xxi, 847 pages.
9. Cervino C, Weber E, Knopp D, & Niessner R (2008) Comparison of hybridoma screening methods for the efficient detection of high-affinity hapten-specific monoclonal antibodies. *J Immunol Methods* 329(1-2):184-193.
10. Covance Inc. www.covance.com/content/dam/covance/assetLibrary/salessheets/60daymonoclonalantibssimmu011-0412spdf.pdf (accessed May. 11, 2020).
11. Clackson T, Hoogenboom HR, Griffiths AD, & Winter G (1991) Making Antibody Fragments Using Phage Display Libraries. *Nature* 352(6336):624-628.
12. Fuchs P, Breitling F, Dubel S, Seehaus T, & Little M (1991) Targeting Recombinant Antibodies to the Surface of Escherichia-Coli - Fusion to a Peptidoglycan Associated Lipoprotein. *Bio-Technol* 9(12):1369-1372.
13. Boder ET & Wittrup KD (1997) Yeast surface display for screening combinatorial polypeptide libraries. *Nat Biotechnol* 15(6):553-557.
14. Lee CMY, Iorno N, Sierro F, & Christ D (2007) Selection of human antibody fragments by phage display. *Nature Protocols* 2(11):3001-3008.
15. Vaughan TJ*, et al.* (1996) Human antibodies with sub-nanomolar affinities isolated from a large non-immunized phage display library. *Nat Biotechnol* 14(3):309-314.
16. Adler AS*, et al.* (2018) A natively paired antibody library yields drug leads with higher sensitivity and specificity than a randomly paired antibody library. *MAbs* 10(3):431-443.
17. Adler AS*, et al.* (2017) Rare, high-affinity mouse anti-PD-1 antibodies that function in checkpoint blockade, discovered using microfluidics and molecular genomics. *MAbs* 9(8):1270-1281.
18. Adler AS*, et al.* (2017) Rare, high-affinity anti-pathogen antibodies from human repertoires, discovered using microfluidics and molecular genomics. *MAbs* 9(8):1282-1296.
19. Rajan S*, et al.* (2018) Recombinant human B cell repertoires enable screening for rare, specific, and natively paired antibodies. *Commun Biol* 1:5.

20. Egloff P, *et al.* (2019) Engineered peptide barcodes for in-depth analyses of binding protein libraries. *Nat Methods* 16(5):421-428.
21. Love JC, Ronan JL, Grotenbreg GM, van der Veen AG, & Ploegh HL (2006) A microengraving method for rapid selection of single cells producing antigen-specific antibodies. *Nat Biotechnol* 24(6):703-707.
22. Jia B, *et al.* (2017) Longitudinal multiparameter single-cell analysis of macaques immunized with pneumococcal protein-conjugated or unconjugated polysaccharide vaccines reveals distinct antigen specific memory B cell repertoires. *Plos One* 12(9):e0183738.
23. A. B. Inc., https://www.abcellera.com/about, (accessed June. 13, 2019)
24. Chen B, *et al.* (2016) High-throughput analysis and protein engineering using microcapillary arrays. *Nat Chem Biol* 12(2):76-81.
25. ©xCellaBiosciences®, https://www.xcellabio.com/, (accessed June. 13, 2019).
26. S. F. Inc., Watch Cyto-Mine® in Action, https://spherefluidics.com/cyto-mine-in-action/?v=7516fd43adaa, (accessed June. 13 2019).
27. Mettler Izquierdo S, *et al.* (2016) High-efficiency antibody discovery achieved with multiplexed microscopy. *Microscopy (Oxf)* 65(4):341-352.
28. Winters A, *et al.* (2019) Rapid single B cell antibody discovery using nanopens and structured light. *MAbs* 11(6):1025-1035.
29. Parola C, Neumeier D, & Reddy ST (2018) Integrating high-throughput screening and sequencing for monoclonal antibody discovery and engineering. *Immunology* 153(1):31-41.
30. Gerard A, *et al.* (2020) High-throughput single-cell activity-based screening and sequencing of antibodies using droplet microfluidics. *Nat Biotechnol*.
