# Supplementary material for: Rapid isolation of antigen-specific B-cells using droplet microfluidics
Source: RSC Adv. 2020 Jul 20;10(45):27006–13. doi: 10.1039/d0ra04328a (PMC9055518; doi:10.1039/d0ra04328a)
Supplement: RA-010-D0RA04328A-s004 [file RA-010-D0RA04328A-s004.pdf]

Electronic Supplementary Material (ESI) for RSC Advances.
This journal is © The Royal Society of Chemistry 2020

Supplementary Information Dataset 2: Sequences of constructs used for cell-free synthesis of scFv proteins. Key sequence elements are indicated by color-coded highlighting. The scFv numbers correspond to the numbers in Figure 3.

T7 promotor First ATG VH-VL Linker Myc sequence

scFv #1
GCGAATTTAATACGACTCACTATAGGGCTTAAGTAAAGGAGGAAAAAATATGA
GTAACAAAAAAACAACAGCAGGTGGTCTCGAGGGTGGTGGTTCTGAGGTAG
AGCTGGTGGAGTCTGGGGGCGGCTTAGTGCAGCCTGGAAGGTCCATGAAACT
CTCCTGTGCAGCCTCAGGATTCACTTTCAGTAATTTTGGCATGGCCTGGGTCC
GCCAGGCTCCAACGAAGGGTCTGGAGTGGGTCGCATCCATTAGTACTAGTGG
TGGTAACACTTACTATCGAGACTCCGTGAAGGGCCGATTCACTATCTCCAGA
GATAATGCAAAAAGCACCCTATACCTGCAAATGGACAGTCTGAGGTCTGAGG
ACACGGCCACTTATTACTGTACAACTCTAGATATAACAGTGGTCGCCTACTTT
GATTACTGGGGCCAAGGAGTCATGGTCACAGTCTCCTCAGCTGAAACGGGCG
GCGGAGGTAGCGGTGGTGGAGGAAGCGGAGGAGGCGGGTCCGACATTGTGC
TGACCCAGTCTCCTGCTTTGGCTGTGTCTCTAGGGCAGAGGGCCACCATCTCT
TGCAAGACCAACCAGGATGTCGATTATTATGGCAATAGTTATATACACTGGT
ACCAACAGAAACCAGGGCAACAACCCAAACTCCTCATCTTTTTAGTATCCAA
CTTAGCATCTGGGATCCCTGCCAGGTTCAGTGGTAGAGGGTCTGGGACAGAC
TTCACCCTCACCATTGATCCTGTGGAGGCTGATGATGCTGCAACCTATTACTG
TCAGCAGAGTAGGAATCTTCCGTACACGTTTGGAGCTGGGACCAAGCTGGAA
CTGAAACGGGCTGATGGTGGAGGTAGCGAGCAAAAGTTGATTTCCGAAGAG
GACTTGTAACTAGCATAACCCCTCTCTAAACGGAGGGGTTT

scFv #2
GCGAATTTAATACGACTCACTATAGGGCTTAAGTAAAGGAGGAAAAAATATG
AGTAACAAAAAAACAACAGCAGGTGGTCTCGAGGGTGGTGGTTCTCAG
GTACAGCTGCAGCAGTCTGGGGGAGGCTTAGTGCAGCCTGGAAGGTCC
CTGAAACTCTCCTGTGCAGCCTCAGGATTCACTTTCAGTGACTATTACA
TGGCCTGGGTCCGCCAGGCTCCAAAGAAGGGTCTGGAGTGGGTCGCAT
CCATTAGTTATGAGGGTAGTAGCACTTACTATGGAGACTCCGTGAAGG
GCCGATTCACTATCTCCAGGGATAATGCAAAAAGCACCCTATACCTGC
AAATGAACAGTCTGAGGTCTGAGGACACGGCCACTTATTATTGTGCAA
GACATCTTGCGTATACTACGGATTCCCCGCTTATGGACGCCTGGGGTCA
AGGAGCTTCAGTCACTGTCTCGAGTGCTAAAACAGGCGGCGGAGGTAG
CGGTGGTGGAGGAAGCGGAGGAGGCGGGTCCGACATTGTCTTGACCCA
GTCTCCTTCACTCCTGTCTGCACCTGTGGGAGACAGAGTCACTCTCAAC
TGCAAAGCAAGTCAGAATATTTATAAGAACTTAGCCTGGTATCAGCAA
AAGCTTGGAGAAGCTCCCAAACTCCTGATTTATAATGCAAACAGTTTG
CAAACGGGCATCCCATCAAGGTTCAGTGGCAGTGGATCTGGTACAGAT
TTCACACCCACCATCAGCAGCCTGCAGCCTGAAGATGTTGCCACATATT
TCTGCCAGCAGTATTATAGCGGGTACACGTTTGGAGCTGGGACCAAGC

TGGAATTGAAACGTACGGATGGTGGAGGTAGCGAGCAAAGTTGATTTCC
GAAGAGGACTTGTAACTAGCATAACCCCTCTCTAAACGGAGGGGTTT

scFv #3
GCGAATTTAATACGACTCACTATAGGGCTTAAGTAAAGGAGGAAAAAATATGA
GTAACAAAAAAACAACAGCAGGTGGTCTCGAGGGTGGTGGTTCTCAGGTGCA
GCTGATGGAGTCTGGGGGAGGCTTAGTGCAGCCTGGAAGGTCCATGAAACTC
TCCTGTGCAGCCTCAGGATTCACTTTCACTAACTATTACATGGCCTGGGTCCG
CCAGGCTCCAACGAAGGGTCTGGAGTGGGTCGCATCCATTAGTACTGGTGGT
GATGTCACTTACTCTCGAGACTCCGTGAGGGGCCGATTCACTATCTCCAGAGA
TAATGCAAAAAGCACCCTATATATGCAAATGGACAGTCTGAGGTCTGAGGAC
ACGGCCACTTATTACTGTGCAAGACGGCGCTACGGTACCTCGGACTACTTTGA
TTACTGGGGCCAAGGAGTCATGGTCACAGTCTCGAGTGCTAAAACAGGCGGC
GGAGGAAGCGGAGGAGGCGGGTCCGACATCCAGATGACCCAGTCTCCTGCCT
CCCTGTCTGCTTCTCTGGAAGAAATTGTCACCATCACCTGCAAGGCAAGCCA
GGATATTGATGATTACTTATCATGGTATCAGCAGAAACCAGGGAAATCTCCT
CAGCTCCTGATCTATGATGCAACCAGTTTGGCAGATGGGGTCCCATCACGGTT
CAGCGGCAGTAGATCTGACACACAGTATTCTCTTAAGATCAGCAGACCACAG
GTTGATGATTCTGGAATCTATTACTGTCTACAGAGTCACAGTATTCCGTGGAC
GTTCGGTGGCGGCACCAAGCTGGAAATAAAACGTACGGATGGTGGAGGTAG
CGAGCAAAAGTTGATTTCCGAAGAGGACTTGTAACTAGCATAACCCCTCTCT
AAACGGAGGGGTTT

scFv #4
GCGAATTTAATACGACTCACTATAGGGCTTAAGTAAAGGAGGAAAAAATATGA
GTAACAAAAAAACAACAGCAGGTGGTCTCGAGGGTGGTGGTTCTGAGGTGCA
CCTGGTGGAGTCTGGGGGAGGCTCAGTGCAGCCTGGAAGGTCCATGAAACTC
TCCTGTGCAGCCTCAGGATTCACTTTCAGTAACTATTACATGGCCTGGGTCCG
CCAGGCTCCAACGACGGGTCTGGAGTGGGTCGCATCCATCAGTACTGGTGGT
GGTAACACTTATTATCGAGACTCCGTGAAGGGCCGATTCACTATTTCCAGAG
ATAATGCACAAAACACCCTTTACCTGCAGATGGACAGTCTGAGGTCTGAGGA
CACGGCCACTTATTACTGTGCAGGGGGGGACCCGGGTATAACCCCTGATTAC
TGGGGCCAAGGAGTCACGGTCACAGTCTCGAGTGCTAAAACAGGCGGCGGA
GGTAGCGGAGGAGGCGGGTCCGACATCCAGATGACCCAGACTCCAACAACC
ATGGCTGCATCTCCAGGAGAGAAGGTCACCATCACCTGCCGTGCCAGCTCAA
GTGTAAGCTACATGCACTGGTTCCAGCAGAAGTCAGGCACCTCCCCCAAACC
CTGGATTTATGACACATCCAAGCTGGCTTCTGGAGTCCCAGATCGCTTCAGTG
GCAGTGGGTCTGGGACCTCTTATTCTCTCACAATCAGCTCCATGGAGGCTGAA
GATGCTGCTACTTATTACTGTCTGCAGAGGAGTAGTTACCCGTACACGTTTGG
AGCTGGGACCAAGCTGGAAATAAAACGTACGGATGGTGGAGGTAGCGAGCA
AAAGTTGATTTCCGAAGAGGACTTGTAACTAGCATAACCCCTCTCTAAACGG
AGGGGTTT

scFv #5
GCGAATTAATACGACTCACTATAGGGCTTAAGTAAAGGAGGAAAAAATATGA
GTAACAAAAAAACAACAGCAGGTGGTCTCGAGGGTGGTGGTTCTCAGGTTCA
TCTGCAACAGTCTGGGGCTGCACTGGTGAAGCCTGGGGCCTCTGTGAAGTTG
TCTTGCAAAGCTTCTGGTTATACATTCACTGACTACTATATACACTGGGTGAA
GCAGAGTCATGGAAAGAGCCTTGAGTGGATTGGGTATATTAATCCTAACAGT
GGTTATACTAACTACAATGAAAAGTTCAAGAGCAAGGCCACATTGACTGTAG
ACAAATCCACCAATACAGCCTATATGGAGCTTAGCAGATTGACATCTGAGGA
CTCTGCAACCTATTACTGTACAAGAGAGGGGTCTGACTACAGTGGTCCGTTTG
CTTACTGGGGCCAAGGCACTCTGGTCACTGTCTCGAGTGCTAAAACAGGCGG
CGGAGGTAGCGGTGGTGGAGGAAGCGGAGGAGGCGGGTCCGACATCCAGAT
GACACAGTCTCCCACATCTATGTTCACATCAGTAGGAGACAGGGTTACCATG
AGCTGCAAGGCCAGTCAGAATGTAGGTATTAATGTAGGCTGGTACCAACAGA
AAACAGGGCAGTCTCCTAAACGGCTTATCTACTGGGCATCCAACCGGGACAC
TGGGGTCCCTGATCGCTTCACAGGTAGTGGATCTGGGACAGATTTCACTCTCA
CCATCAGCAACATGCAGGCTGAAGACCCGGCTATTTATTACTGTCTGCAGCAT
AACTCCTATCCGCTCACGTTCGGTTCTGGGACCAAGCTGGAAATAAAACGTA
CGGATGGTGGAGGTAGCGAGCAAAAGTTGATTTCCGAAGAGGACTTGTAACT
AGCATAACCCCTCTCTAAACGGAGGGGTTT

scFv #6
GCGAATTAATACGACTCACTATAGGGCTTAAGTAAAGGAGGAAAAAATATGA
GTAACAAAAAAACAACAGCAGGTGGTCTCGAGGGTGGTGGTTCTGAGGTGA
AGCTGGTGGAGTCTGGCCCTGGGATATTGCAGCCCTCCCAGACCCTCAGTCT
GACTTGCACTTTCTCTGGGTTTTCACTGAGCACTTATAGTATGGGTGTGGGCT
GGATTCGTCAGCCTTCAGGGAAGGGTCTGGAGTGGCTGGCAAACATTTGGTG
GGATGATGATAAGTTCTCCCATCCATCTCTGAAAAACCGGCTCACAATCTCCA
AGGACACCTCCAACAACCAAGCATTCCTCAAGATCACCAATGTGGACACTGC
AGATACTGCCACATACTACTGTACTCGGATCAGAGGTAACAACTACGCGGGG
GACTACTTTGATTACTGGGGCCAAGGAGTCATGGTCACAGTCTCGAGTGCTA
AAACAGGCGGCGGAGGTAGCGGTGGTGGAGGAAGCGGAGGAGGCGGGTCCG
ACATCCAGATGACACAGTCTCCTTCACTCCTGTCTGCATCTGTGGGAGACAGA
GTCACTCTCAGCTGCAAAGGAAGTCAGAATATTCACAATTATTTAGCCTGGTA
CCAACAAAGGCTTGGAGAAGCTCCCAAACTCCTGATACATAAAACAAACAGT
TTGCAAACGGGCATCCCATCAAGGTTCAGTGGCAGTGGATCTAATACAGATT
ACACACTCACCATCAGCAGCCTGCACTCTGAAGATCTTGCCACATATTACTGC
TATCAGTATTACAACGGGCTCACGTTCGGTTCTGGGACCAAGCTGGAAATAA
AACGTACGGATGGTGGAGGTAGCGAGCAAAAGTTGATTTCCGAAGAGGACTT
GTAACTAGCATAACCCCTCTCTAAACGGAGGGGTTT

scFv #7
GCGAATTTAATACGACTCACTATAGGGCTTAAGTAAAGGAGGAAAAAATATGA
GTAACAAAAAAACAACAGCAGGTGGTCTCGAGGGTGGTGGTTCTGAGGTGCA
CCTGGTGGAGTCAGGACCTGGTCTGGTGCAGCCCTCAGAGACCCTGTCCCTC
ACCTGCACTGTCTCTGGGTTCTCACTAACCAGCTATAGTGTAAGTTGGGTTCG
CCAGCCTTCAGGAAAAGGTCCTGAGTGGATGGGAAGAATGTGGTATGATGGA
GACACAGCATATAATTCAGCTCTCAAATCCCGACTGAGCATCAGCAGGGACA
CCTCCAAGAACCAAGTTTTCTTAAAAATGAACAGTCTGCAAACTGATGACAC
AGGCACTTACTACTGTACCAGAGATCGCTCGGCCAAACTACGGAGGGTCGCC
CGCAGGGAGTCACGGTCACAGTCTCGAGTGCTAGGCGGCGGAGGTAGCGGTG
GTGGAGGAAGCGGAGGAGGCGGGTCCGACATCCAGTTGACCCAGTCTCCAG
CTTCCCTGTCTGCATCTCTGGGAGAAACTGTCACCATCGAATGTCGAGCAAGT
GAGGACATTTACAGTAATTTAGCGTGGTATCAGCAGAAACCAGGGAACTCTC
CTCAGCTCCTGATCTATGATGCAAATAGCTTGGCAGATGGGGTCCCATCACG
GTTCAGTGGCAGTGGATCTGGCACACAGTATTCTCTAAAGATAAACAGCCTG
CAATCTGAAGATGTCGCAAGTTATTTCTGTCAACGGTATAACAATTATCCTCC
CACGTTTTGA*GCTGGGACCAAGCTGGAATTGAAACGTACGGATGGTGGAGG
TAGCGAGCAAAAGTTGATTTCCGAAGAGGACTTGTAACTAGCATAACCCCTC
TCTAAACGGAGGGGTTT
*: In-frame stop codon.

scFv #8
GCGAATTTAATACGACTCACTATAGGGCTTAAGTAAAGGAGGAAAAAATATGA
GTAACAAAAAAACAACAGCAGGTGGTCTCGAGGGTGGTGGTTCTGAGGTGCA
GCTGCAGGAGTCTGGACCTGGCCTGGTGCAGCCCTCACAGACCCTGTCTCTC
ACCTGCACTGTCTCTGGGTTCTCATTAACCAGCTATCATGTGCACTGGGTTCG
ACAGCCTCCAGGGAAAGGTCTGGAGTGGATGGGAGTAATGTGGAGTGATGG
AGACACATCATATAATTCAGCTCTCAAATCCCGACTGAGCATCAGCAGGGAC
ACCTCCAAGAGCCAAGTTTTCTTAAAAATGAGCAGTCTGCAAACTGAAGACA
CAGCCACTTACTACTGTGCCAGAGATCCCTTTATAACAACTACGGACTTTGAT
TACTGGGGCCAAGGAGTCACGGTCACAGTCTCGAGTGCTAAAACAGGCGGCG
GAGGTAGCGGTGGAGGCGGGTCCGATGTTTTGATGACACAAAGTCCATTCTC
CCTGGCTGTGTCAGAAGGAGAGATGGTCACTATAAACTGCAAGTCCAGTCAG
AGTCTTTTATCCAGTGGAAACCAAAAGAAGTACTTGGCTTGGTACCAGCAGA
AACCAGGGCAGTCTCCTAAACTACCGATCTACTATGCATACACTAGGCAATC
AGGGGTCCCTGATCGCTTCATAGGCAGTGGATCTGGGACAGACTTCACTCTG
ACCATCAGCGATGTGCAGGCTGAAGACCTGGCAGATTATTACTGCCTGCAGC
ATTACAGTTCTCCGTACACGTTTGGAGCTGGGACCAAGCTGGAAATAAAACG
TACGGATGGTGGAGGTAGCGAGCAAAAGTTGATTTCCGAAGAGGACTTGTAA
CTAGCATAACCCCTCTCTAAACGGAGGGGTTT

scFv #9

GCGAATTTAATACGACTCACTATAGGGCTTAAGTAAAGGAGGAAAAAATATGA
GTAACAAAAAAACAACAGCAGGTGGTCTCGAGGGTGGTGGTTCTGAGGTGCA
GCTGCAGGAGTCAGGACCTGGCCTGGTGCAACCAACACAGACCCTGTCCATC
ACATGTACTGTTTCTGGGTTCTCATTAACCAGCTATTATATGCAGTGGGTTCG
CCAGACTCCAGGAAAGGGACTAGAATGGATGGGATTTATACGGAGTGGTGG
AAGCACAGAGTATAATTCAGAGTTCAAATCCCGACTTAGCATCAGCAGGGAC
ACCTCCAAGAACCAAGTTTTCTTAGAAATGAACAGTCTGAAAACAGAGGACA
CAGGCGTGTACTACTGTGCCAGAGCGGCTATAGCAGCTATATCCCTTATGGAT
GCCTGGGGTCAAGGAGCTTCAGTCACTGTCTCGAGTGCTAAAACAGGCGGCG
GAGGTAGCGGTGGTGGAGGAAGCGGAGGAGGCGGGTCCGATGTTGAGCTGA
CCCAGACTCCATCTTATCTTGCTGCGTCTCCTGGAGAAAGTGTTTCCATCAAT
TGCAAGGCAAGTAAAAGCATTAACACATACTTAGCCTGGTATCAGGAGAAAC
CTGGGAAAACGAATAAGCTTCTTATCTACTCTGGGTCAACTTTGCAATCTGGA
ACTCCATCGAGATTCAGTGGCAGTGGATCTGGTACAGATTTCACGCTCACCAT
CAGAAGCCTGGAGCCTGAAGATTTTGCAGTCTACTACTGTCAACAGCATAAT
GAATACCCGCTCACGTTCGGTTCTGGGACCAAGCTGGAAATAAAACGTACGG
ATGGTGGAGGTAGCGAGCAAAAGTTGATTTCCGAAGAGGACTTGTAACTAGC
ATAACCCCTCTCTAAACGGAGGGGTTT

scFv #10

GCGAATTTAATACGACTCACTATAGGGCTTAAGTAAAGGAGGAAAAAATATGA
GTAACAAAAAAACAACAGCAGGTGGTCTCGAGGGTGGTGGTTCTGAAATGCA
GCTGGTGGAGTCTGGGGGAGGCCTAGTGCAGCCTGGAAGGTCCCTGAAACTC
TCCTGTGCAGCCTCAGGATTCACTTTCAGTAACTATTACATGGCCTGGGTCCG
CCAGGCTCCAAAGAAGGGTCTGGAGTGGGTCGCAACCATTAGTACCAGTGGT
AGCAGAACTTACTATCCAGACTCCGTGAAAGGCCGATTCACTATCTCCAGAG
ATAATGCAAAAAGCAGCCTATACCTGCAAATGAACAGTCTGAAGTCTGAGGA
CACGGCCACTTATTACTGTGCAAGAGGATCAACTACGAATTGGTTTGCTTACT
GGGGCCAAGGCACTCTGGTCACTGTCTCGAGTGCTAAAACAGGCGGCGGAGG
TAGCGGTGGTGGAGGAAGCGGAGGAGGCGGGTCCGACATCCAGATGACCCA
GTATCCTTCACTCCTGTCTGCATCTGTGGGAGACAGAGTCACTCTTAGCTGCA
AAGGAAGTCAGAATATTAACAATTACTTAGCCTGGTACCAACAAAAGCTTGG
AGAAGCTCCCAAACTCCTGATATATAATACAAACAGTTTGCAAACGGGCATC
CCATCAAGGTTCAGTGGCAGTGGATCTGGTACAGATTACACACTCACCATCA
GCAGCCTGCAGCCTGAAGATGTTGCCACATATTTCTGCTATCAGTATAACAAC
GGGTACACGTTTGGAGCTGGGACCAAGCTGGAACTGAAACGTACGGATGGTG
GAGGTAGCGAGCAAAAGTTGATTTCCGAAGAGGACTTGTAACTAGCATAACC
CCTCTCTAAACGGAGGGGTTT

scFv #11

GCGAATTTAATACGACTCACTATAGGGCTTAAGTAAAGGAGGAAAAAATATGA
GTAACAAAAAAACAACAGCAGGTGGTCTCGAGGGTGGTGGTTCTGAGGTGA
AGCTGGAGGAGTCTGGGGGAGGCTTAGTGCAGCCTGGAAGGTCCCTGAAACT
CTCCTGTGCAGCCTCAGGATTCACTTTCAGTAACTATGACATGGCCTGGGTCC
GCCAGGCTCCAACGAAGGGTCTGGAGTGGGTCGCATCCATTAGTCCTAGTGG
TGGTAGCACTTACTATCGAGACTCCGTAAAGGGCCGATTCACTGTCTCCAGA
GATAATGCAAAAAGCAGCCTATACCTGCAAATGGACAGTCTGAGGTCTGAGG
ACACGGCCACTTATTACTGTGCAAGACATAAGAATCCCGGGTATAACCAGGA
TTACTGGGGCCAAGGAGTCACGGTCACAGTCTCGAGTGCTAAAACAGGCGGC
GGAGGTAGCGGTGGTGGAGGAAGCGGAGGAGGCGGGTCCGACGTTGTGAT
GACCCAGACTCCATCCTCCCAGGCTGTGTCAGCAGGGGAGAAGGTCAC
TATGAGCTGCAAGTCCAGTCAGAGTCTTTTATACAATGAAAACAAAAA
GAACTACTTGGCCTGGTACCAGCAGAAACCAGGGCAGTCTCCTAAACT
GCTGATCTACTGGGCATCCACTAGGGAATCTGGGGTCCCTGATCGCTTC
ATAGGCAGTGGATCTGGGACAGATTTCACTCTGACCATCAGCAGTGTG
CAGGCAGAAGACCTGGCTGTTTATTACTGCCAGCAGTACTATAACTTTC
CTCGGACGTTCGGTGGAGGCACCAAGCTGGAAATAAAACGTACGGATG
GTGGAGGTAGCGAGCAAAAGTTGATTTCCGAAGAGGACTTGTAACTAGCATA
ACCCCTCTCTAAACGGAGGGGTTT

scFv #12

GCGAATTTAATACGACTCACTATAGGGCTTAAGTAAAGGAGGAAAAAATATGA
GTAACAAAAAAACAACAGCAGGTGGTCTCGAGGGTGGTGGTTCTGAGGTGCA
GTTGGTGGAGTCTGATGGAGGCTTAGTGCAGCCTGGAAGGTCCCTAAAACTC
TCCTGTGCAGCCTCAGGATTCACTTTCAGTGACTATTACATGGCCTGGGTCCG
CCAGGCTCCAACGAAGGGGCTGGAGTGGGTCGCAACCATTAGTTATGATGGT
AGTAGCACTTACTATCGAGACTCCGTGAAGGGCCGATTCACTATCTCCAGAG
ATAATGCAAAAAGCACCCTATACCTGCAAATGGACAGTCTGAGGTCTGAGGA
CACGGCCACTTATTACTGTGCAAGAACGGATAGCAGCTATATCTCTGATTACT
GGGGCCAAGGAGTCACGGTCACAGTCTCGAGTGCTAAAACAGGCGGCGGAG
GTAGCGGTGGTGGAGGAAGCGGAGGAGGCGGGTCCAACATCCAGATGACCC
AGTCTCCATCCTCTCTGGCTGTGTCAGCAGGAGAGACGGTCACTATAAACTGC
AAGTCCAGTCAGAGTCTTTTATACAGTGGAAACCAAAAGAACTACTTGGCCT
GGTACCAGCAGAAACCAGGGCAGTCTCCTAAACTGCTGATCTACTGGGCATC
TACTAGGCAATCTGGTGTCCCTGATCGCTTCATAGGCAGTGGATCTGGGACA
GACTTCACTCTGACCATCAGCAGTGTGCAGGCAGAAGATCTGGCAATTTATT
ACTGTCAGCAGTATTATGATACTCCATTCACGTTCGGCTCAGGGACGAAGTTG
GAAATAAAACGTACGGATGGTGGAGGTAGCGAGCAAAAGTTGATTTCCGAA
GAGGACTTGTAACTAGCATAACCCCTCTCTAAACGGAGGGGTTT

scFv #13

GCGAATTTAATACGACTCACTATAGGGCTTAAGTAAAGGAGGAAAAAATATGA
GTAACAAAAAAACAACAGCAGGTGGTCTCGAGGGTGGTGGTTCTGAGGTGCA
GCTGGTGGAGTCTGGGGGAGGCTTAGTGCAGCCTGGAAGGTCCATGAAACTC
TCCTGTGCAGCCTCAGGATTCACTTTCAGCAACTATGACATGGCCTGGGTCCG
CCAGGCTCCAAAGAAGGGTCTGGAGTGGGTCGCAACCATTAGTTATGATGGT
AGTAGCACTTACTTTCGAGACTCCGTGAAGGGCCGATTCACTATCTCCAGAG
ATAATGAAAAAAGCACCCTATACCTGCAAATGGACGGTCTGAGGTCTGAGGA
CACGGCCACTTATTACTGTACAACACGGGGGATTACCTTCCCGCACTACTTTG
ATTACTGGGGCCAAGGAGTCATGGTCACAGTCTCCTCAGCTGAAGGCGGCGG
AGGTAGCGGTGGTGGAGGAAGCGGAGGAGGCGGGTCCGATGTTGTGCTGAC
CCAGACTCCACCCACTTTGTCGGCTACCATTGGACAATCAGTCTCCATCTCTT
GCAGGTCAAGTCAGAGTCTCTTACATAGTAATGGAAACACCTATTTTCATTGG
TTACTACAGAGGCCAGGCCAATCTCCACAGCTTCTAATTCACTTGGTATCCAG
ACTGGAATCTGGGGTCCCCAACAGGTTCAGTGGCAGTGGGTCAGGAACTGAT
TTCACACTCAAAATCAGTGGAGTAGAGGCTGAGGATTTGGGAGTTTTTTATTG
TATACAAGGTACCCATGCTCCGTGGACGTTCGGTGGAGGCACCAGGCTGGAA
TTGAAACGGGCTGATGGTGGAGGTAGCGAGCAAAAGTTGATTTCCGAAGAGG
ACTTGTAACTAGCATAACCCCTCTCTAAACGGAGGGGTTT

scFv #14

GCGAATTTAATACGACTCACTATAGGGCTTAAGTAAAGGAGGAAAAAATATGA
GTAACAAAAAAACAACAGCAGGTGGTCTCGAGGGTGGTGGTTCTGAGGTGCA
GCTGGTGGAGTCTGGAGGAGGCTTAGTGCAGCCTGGAAGGTCCCTGAAACTC
TCCTGCTTAGCCTCTGGATTCACTTTCAGTAACTATGGAATGAACTGGATTCG
CCAGGCTCCAGGGAAGGGGCTGGAGTGGGTTGCATCTATTAGTAGTAGTAGC
AGTTACATCTACTATGCAGACACAGTGAAGGGCCGATTCACCATCTCCAGAG
AAAATGCCAAGAACACCCTGTACCTGCAAATGACCAGTCTGAGGTCTGAAGA
CACTGCCTTGTATTACTGTGCAAGAGAGGGCCGGGGGGCTATGGATGCCTGG
GGTCAAGGAACTTCAGTCACTGTCTCCTCAGCTGAAGGCGGCGGAGGTAGCG
GTGGTGGAGGAAGCGGAGGAGGCGGGTCCGACATTCAGATGACCCAGTCTCC
ATCCTCCATGTCTGTGTCTCTGGGAGACACAGTCACTATTACTTGCCGGGCAA
GTCAGGACGTTGGGATTTATGTAAACTGGTTCCAGCAGAAACCAGGGAAATC
TCCTAGGCGTATGATTTATCGTGCAACGAACTTGGCAGATGGGGTCCCATCA
AGGTTCAGCGGCAGTAGGTCTGGATCAGATTATTCTCTCACCATCAGCAGCCT
GGAGTCTGAAGATGTGGCAGACTATCACTGTCTACAGTATGATGAGTATCCG
TACACGTTTGGAGCTGGGACCAAGCTGGAACTGAAACGGGCTGATGGTGGAG
GTAGCGAGCAAAAGTTGATTTCCGAAGAGGACTTGTAACTAGCATAACCCCT
CTCTAAACGGAGGGGTTT

scFv #15

GCGAATTTAATACGACTCACTATAGGGCTTAAGTAAAGGAGGAAAAAATATGA
GTAACAAAAAAACAACAGCAGGTGGTCTCGAGGGTGGTGGTTCTGAGGTGCA
GCTGGTGGAGTCTGGGGGAGGCTTAGTGCAGCCTGGAAGGTCCATGAAACTC
TCCTGTGCAGCCTCAGGATTCACTTTCAGTAACTATTACATGGCCTGGGTCCG
CCAGGCTCCAACGAAGGGTCTGGAGTGGGTCGCATCCATTAGTACTGGTGGT
GGTAACACTTACTATCGAGACTCCGTGAAGGGCCGATTCACTATCTCCAGAG
ATAATGCAAAAAGCACCCTATACCTGCAAATGGACAGTCTGAGGTCTGAGGA
AACGGCCACTTATTACTGTGCAAGACATAGGTATACTACGGATTATTACTACG
GCTGGTACTTTGACTTCTGGGGCCCAGGAACCATGGTCACCGTGTCCTCAGCC
CAAACAGGCGGCGGAGGTAGCGGTGGTGGAGGAAGCGGAGGAGGCGGGTCC
GATGTTGTGATGACCCAGACACCACCATCTTTGTCGGTTGCCATTGGACAGTC
AGTCTCCATCTCTTGCAAGTCAAGTCAAAGCCTCGTAGCTAGTGATAAAAAT
ACATATTTGAATTGGTTTTTACAGAGTCCTGGCCGGTCTCCGAGGCGCCTAAT
CTCTCAGGTGTCTAAGCTGGACTCTGGAGTCCCTGACAGGTTCAGTGGCAGTG
GGTCAGAGAAAGATTTCACACTTAAAATCAGCAGAGTGGAGACTGAAGATCT
GGGAGTTTATTACTGCCTGCAAGCTACACATCTTCCATTCACGTTCGGCTCAG
GGACGAAGTTGGACATGAAACGGGCTGATGGTGGAGGTAGCGAGCAAAAGT
TGATTTCCGAAGAGGACTTGTAACTAGCATAACCCCTCTCTAAACGGAGGGG
TTT

Positive control scFv (Pos)

GCGAATTTAATACGACTCACTATAGGGCTTAAGTAAAGGAGGAAAAAATATGA
GTAACAAAAAAACAACAGCAGGTGGTCTCGAGGGGGGAGGCTCTGGAGGGG
GTTCGGAGGTCCAGCTTGTAGAATCCGGAGGCGGGCTGGTGCAGCCCGGCCG
CAGTCTTCGCTTGAGCTGTGCTGCTTCTGGATTCACGTTCGATGATTATGCTAT
GCACTGGGTCCGCCAAGCACCTGGAAAAGGGCTGGAATGGGTGTCAGCGATT
ACTTGGAATTCGGGGCACATCGATTATGCAGACTCCGTCGAGGGGCGCTTTA
CCATTAGTCGTGATAATGCGAAAAACAGCCTTTACCTTGACATGAATTCGTTA
CGTGCTGAGGATACCGCGGTGTATTACTGTGCAAAGGTGAGCTACTTGAGCA
CCGCCAGTTCACTGGACTACTGGGGACAAGGTACGCTTGTCACCGTTTCGTCA
GCATCAGGCGGCGGAGGTAGCGGGGGTGGGGGAAGCGGAGGGGGCGGGTCC
GATATCCAGATGACCCAAAGTCCTTCATCTCTTAGCGCGTCAGTCGGGGACC
GCGTTACAATCACATGCCGCGCCTCGCAGGGGATCCGTAACTATCTGGCATG
GTACCAGCAGAAACCTGGTAAGGCGCCAAAATTGTTAATTTACGCGGCGTCT
ACACTTCAGAGCGGTGTCCCGTCTCGTTTCAGTGGTTCAGGTTCCGGTACGGA
CTTCACTCTTACAATCTCCTCTTTGCAGCCAGAAGACGTAGCAACATACTACT
GCCAACGCTATAACCGCGCACCATACACGTTCGGTCAAGGGACAAAGGTAGA
GATCAAGCGCACGGGTGGAGGTAGCGAGCAAAAGTTGATTTCCGAAGAGGA
CTTGTAACTAGCATAACCCCTCTCTAAACGGAGGGGTTT

Negative control scFv (Neg)

GCGAATTAATACGACTCACTATAGGGCTTAAGTAAAGGAGGAAAAAATATGA
GTAACAAAAAAACAACAGCAGGTGGTCTCGAGGGTGGTGGTTCTGAGGTGA
AGCTGGAGGAGTCTGGGGGCGGCTTGGTGCAGCCTGGAAGGTCCCTGAAACT
CTCCTGTGCAGCCTCAGGATTCACTTTCAGTAACTATGGCATGCACCGGATCC
GCCAGGCTCCGACGAAGGGTCTGGAGTGGGTCGCATCCATTAGTCCTAGTGG
TGGTAGCACTTACTATCGAGACTCCGTGAAGGGCCGATTCACTATCTCCAGG
GATAATGCAAAAAGCACCCTATACCTGCAAATGGACAGTCTGAGGTCTGAGG
ACACGGCCACTTATTACTGTGCAACAGAGCGGGAGATTACTATAGCAGCTAT
CCCTTACTACTTTGATTACTGGGGCCAAGGAGTCATGGTCACAGTCTCGAGTG
CTAAAACAGGCGGCGGAGGTAGCGGTGGTGGAGGAAGCGGAGGAGGCGGGT
CCGATATTGTGATGACACAGTCTCCAACAACCATGGCTGCGTCTCCTGGGGA
GAAGGTCACCACCACCTGTCATGCCACCTCCAGTGGAATCTACATGAACTGG
CACCAGCAGAAGTCAGGCACCTCTCCCAAACTCTGGATTTATGACACATCCA
GACTGGCTTCTGGAGTCCCAGATCGCTTCAGTGGCAGTGGGTCTGGGACCTCT
TATTCTCTCACAATCAACACCATGGAGACTGAAGATGCTGCCACTTATTATTG
CCAGCAGTGTTGTCCTACCCCGCCCATCACGTTCGGCTCAGGGACGAGGTTG
GAAATAAAACGTACGGATGGTGGAGGTAGCGAGCAAAAGTTGATTTCCGAA
GAGGACTTGTAACTAGCATAACCCCTCTCTAAACGGAGGGGTTT
